# Supplementary material for: A new approach to Health Benefits Package design: an application of the Thanzi La Onse model in Malawi
Source: PLoS Comput Biol. 2024 Sep 30;20(9):e1012462. doi: 10.1371/journal.pcbi.1012462 (PMC11567512; doi:10.1371/journal.pcbi.1012462)
Supplement: S1 Appendix — (DOCX) [file pcbi.1012462.s001.docx]

**Competition for limited HCWs time: understanding rates of service delivery under resource constraints**

***1. Understanding factors affecting the competition for HCWs time***

The resource constraint enforced in our analysis takes the form of HCWs’ time. As discussed in section 2.2, this means that HSIs requiring one or more of the same HCWs at the same facility level will therefore be in direct competition with each other.

We illustrate this for the case of malaria testing in Fig A: malaria tests are delivered either at facility level 0 by Disease Control and Surveillance Assistants (DCSAs) or at facility levels 1a/2 as outpatient appointments, where they require clinical, pharmacy, and nursing officers present. The figure shows all other treatments included in the model which require one (e.g. HIV testing at level 1a), two (e.g. HIV Prevention Circumcision at level 1a) or more of the same HCWs at the relevant levels. This means that the ability of the healthcare system to deliver requested malaria tests will directly limit its ability to deliver all other services listed in this diagram.

The relative prioritisation of those services by different policies will influence the relative probability of certain services winning this competition over others, as discussed in section 2.3. Other factors will however contribute to determine the outcome of this competition:

1. The number of services competing for the same HCW time; the more treatment types a HCW is expected to deliver, the more the competition for its time will be acerbated (see Fig A). As discussed in section 3, policies which deliberately exclude services from their provision therefore have a comparative advantage in delivering low-priority appointments.
2. The relative volume of requests for competing services across different priority levels. Notice that the volume of requests will itself depend on i) use of referral pathways, which will vary for different areas of health, and which will be impacted by the rate of successful treatment delivery along the referral chain, itself affected by the policy under consideration; and ii) on the incidence of the disease in the population, which will itself be affected by the rate of successful treatment delivery both for disease-specific treatments, but also for other treatments linked to relevant risk factors and comorbidities; and iii) on the prob- ability of consumables stock-outs, as patients who fail to receive requested consumables may attempt to receive those consumables again on a subsequent visit;
3. If a treatment is offered at different facility levels (as for the case of malaria discussed above) the relative probability of patients seeking care for that treatment at a level which is more or less affected by competition with other services;
4. The number of HCWs required to perform the treatment: the more officers are required to deliver a treatment, the higher the probability that at least one of them will be out of time;
5. The relative time demands of competing HSIs: treatments with a low volume of requests but which do have a significant time requirement associated with each HSI will still take up a lot of the resources available;
6. Geographical variation in the incidence of different diseases means that the rates of successful treatment delivery may vary district by district.

***2. Understanding the rate of service delivery under different policies***

In the previous section, we discussed how a number of factors will shape the competition for limited HCWs’ time, which makes interpreting the rate of successful treatment delivery under different policies non-trivial. The latter are easiest to untangle at level 0, where only two services are delivered — namely outpatient pneumonia appointments and malaria tests — and only DCSAs are present (as shown in Fig A).

In Fig B we show the mean number of requests for each appointment type at level 0, as well as the fraction of delivered to total requested appointments for all policies considered. For policies which do not enforce any relative prioritisation between malaria tests and pneumonia outpatient appointments (NP, HSSP-III HBP, LCOA, and CMD policies), the rate of treatment delivery for the two services is roughly the same, as expected. For policies which prioritise pneumonia treatments above malaria tests (RMNCH and CV policies), the very low request for this treatment can be satisfied completely by only using a very small fraction of available capabilities, meaning that most of malaria tests, despite having been de-prioritised, can still go ahead (see point 2 in the previous section). On the contrary, policies which prioritise malaria tests, whose request is almost 300 times higher than that for pneumonia appointments at this level, won’t have sufficient capabilities to satisfy the request for malaria tests completely, meaning the probability of malaria test delivery is actually comparable to that of the other policies. Notice however that if this were the whole story, we would expect the rate of outpatient pneumonia treatment delivery to be exactly zero, whereas instead this is closer to 40 %. This due to the fact that strong geographical variations in the incidence of malaria exist in the country, meaning that while level 0 facilities in districts with high incidence of malaria will be completely overwhelmed by that demand — and therefore will struggle to meet it with available capabilities — districts with low incidence of malaria will have much more capabilities to dedicate to pneumonia appointments, increasing the rate of pneumonia treatment delivery (illustrating point 6).

Additional confounding issues in converting the expected return in health from the number of HSIs delivered include the incidence of comorbidities — which will vary policy-by-policy — but also the probability of consumables stock-outs [1], delays in or complete lack of health-seeking in cases where it would be warranted, and imperfect clinical practices (such as diagnostic errors, lack of appropriate referrals and tests), which are all captured statistically by the model, as was illustrated in the case of neonatal disorders previously.

By relying on the TLO simulation, we can however ensure that all such factors are consistently accounted for, such that the resulting rate of HSI delivery under different policies — and its effect on the health burden — is correct.


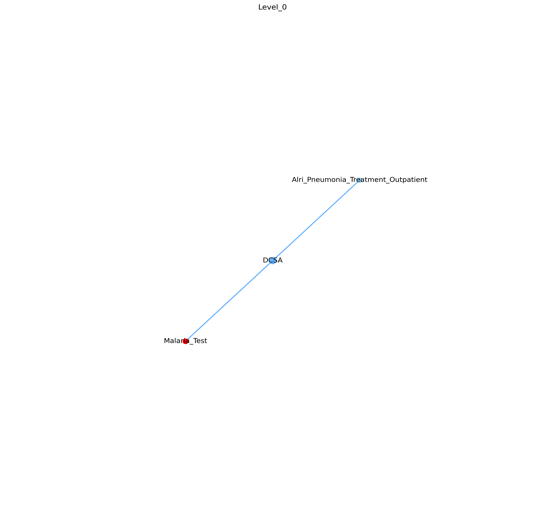

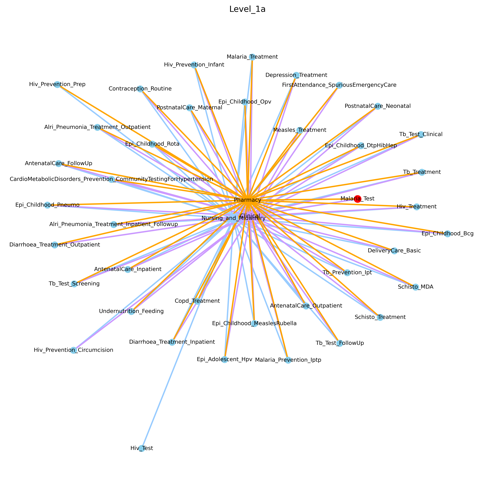

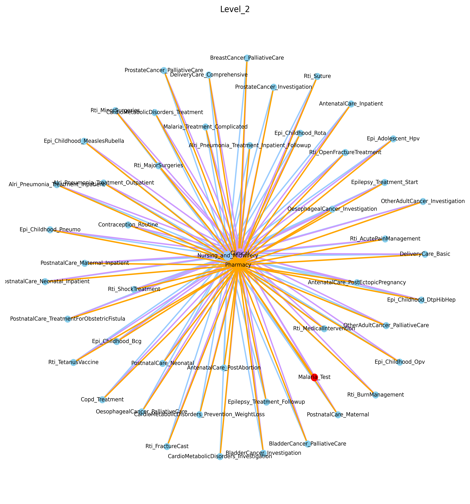


**Fig A**. HSIs competing for the same HCWs as malaria test (shown in red) at different facility levels.


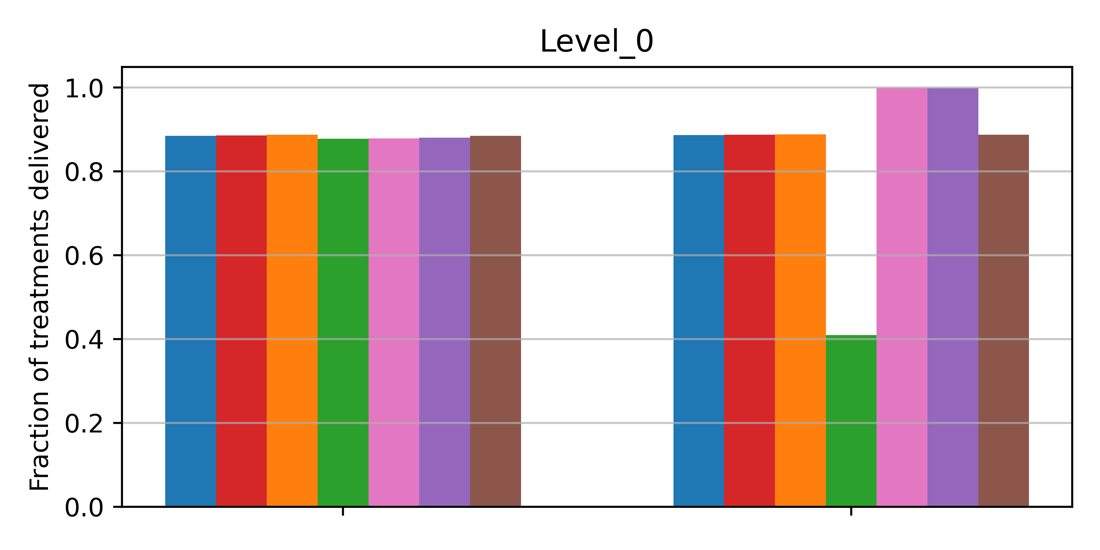

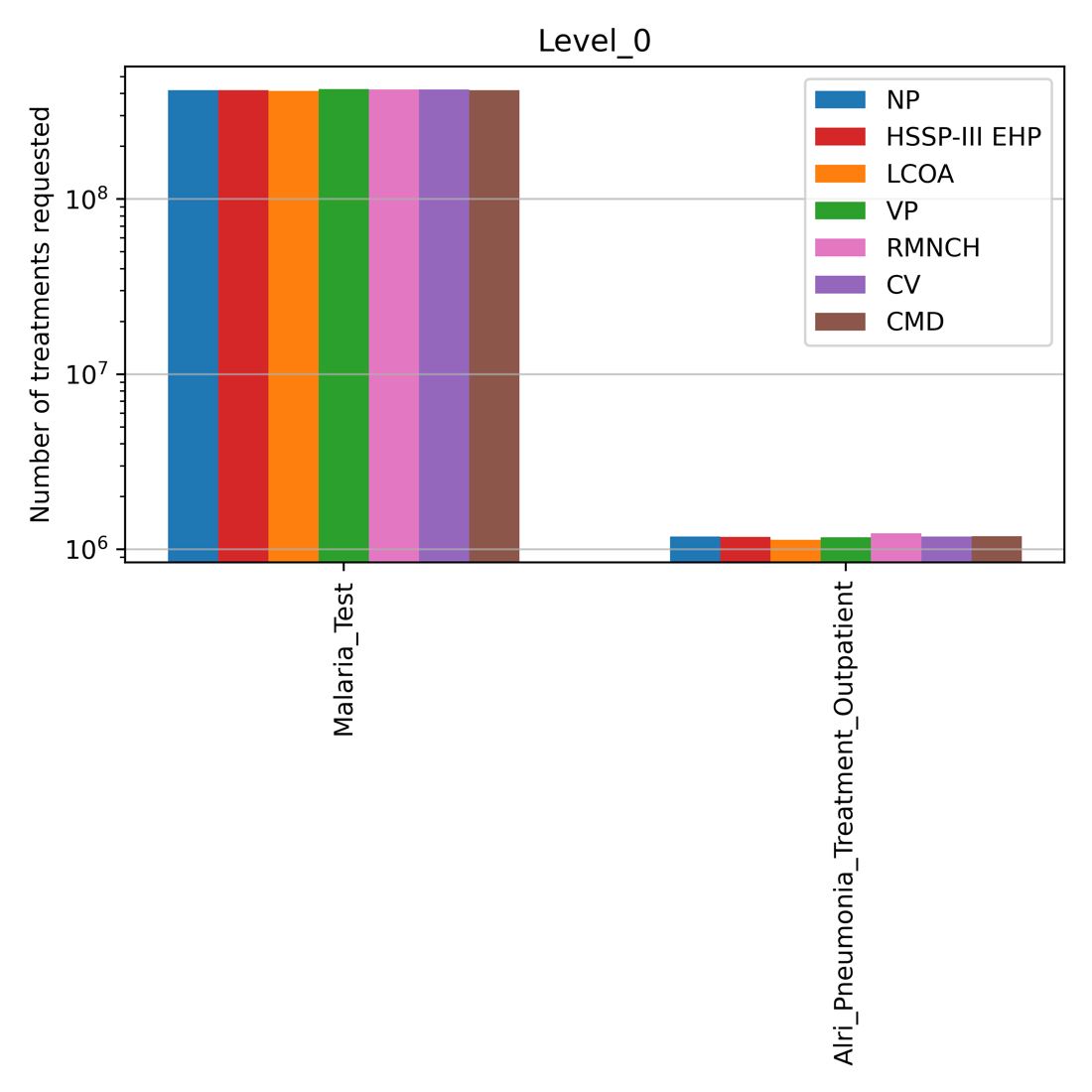


**Fig B**. Mean fraction of total treatments delivered *(top plot)* and mean number of total requests for treatment *bottom plot)* incurred between 2023 and 2042 (inclusive) at facility level 0 under different policies.

**References**

1. Mohan S, Mangal TD, Kadewere G, Chimwaza C, Colbourn T, Collins JH, et al. Factors Associated with Consumable Stock-Outs in Malawi: Evidence from a Facility Census; 2023. Forthcoming. Available from:<http://dx.doi.org/10.2139/ssrn.4424341>.
