# Supplementary material for: A new approach to Health Benefits Package design: an application of the Thanzi La Onse model in Malawi
Source: PLoS Comput Biol. 2024 Sep 30;20(9):e1012462. doi: 10.1371/journal.pcbi.1012462 (PMC11567512; doi:10.1371/journal.pcbi.1012462)
Supplement: S2 Appendix — (PDF) [file pcbi.1012462.s002.pdf]

	LCOA					HSSP-III EHP			
**Treatment ID (TLO Model)**	**Matched interventions (LCOA analysis)**	**Reason for exclusion from LCOA analysis**	**Inclusion recommendation**	**Priority ('LCOA' policy)**	**Further justification of priority assigned**	**Matched interventions (HSSP-III)**	**Inclusion decision**	**Priority ('HSSP-III EHP'' policy)**	**Further justification of priority assigned**
FirstAttendance_Emergency		No matching intervention	NA	0	Emergency	NA	NA	0	Emergency
FirstAttendance_SpuriousEmergencyCare		No matching intervention	NA	0	Emergency	NA	NA	0	Emergency
Alri_Pneumonia_Treatment_Outpatient	Community-based management of neonatal pneumonia		Include	1		Childhood pneumonia treatment (Antibiotics, oxygen, IV fluids - community to secondary level of care)	Include	1	
	Case management of childhood pneumonia		Include			Childhood pneumonia treatment (Antibiotics, oxygen, IV fluids - community to secondary level of care)	Include		
Alri_Pneumonia_Treatment_Inpatient	IMCI Treatment of severe pneumonia	No CE data	NA	2	included with lower priority because no match and for continuity in treatment alongside Alri_Pneumonia_Treatment_Outpatient	Childhood pneumonia treatment (Antibiotics, oxygen, IV fluids - community to secondary level of care)	Include	1	
Alri_Pneumonia_Treatment_Inpatient_Followup	IMCI Treatment of severe pneumonia	No CE data	NA	2	included with lower priority because no match and for continuity in treatment alongside Alri_Pneumonia_Treatment_Outpatient	Childhood pneumonia treatment (Antibiotics, oxygen, IV fluids - community to secondary level of care)	Include	1	
BladderCancer_Investigation		No matching intervention	NA	3	Excluded because tertiary services/ cancer are generally excluded under LCOA	No matching intervention	Exclude	3	
BladderCancer_PalliativeCare		No matching intervention	NA	3	Excluded because tertiary services/ cancer are generally excluded under LCOA	No matching intervention	Exclude	3	
BladderCancer_Treatment		No matching intervention	NA	3	Excluded because tertiary services/ cancer are generally excluded under LCOA	No matching intervention	Exclude	3	
BreastCancer_Investigation	Screening: Mammography		Exclude	3		No matching intervention	Exclude	3	
	Biennial clinical breast examination (CBE) screening + Treatment of breast cancer, stages I-IV		NA			No matching intervention	Exclude		
BreastCancer_PalliativeCare		No matching intervention	NA	3	Excluded because breast cancer interventions are excluded under LCOA	No matching intervention	Exclude	3	
BreastCancer_Treatment	Breast Cancer (first line)		Exclude	3		No matching intervention	Exclude	3	
CardioMetabolicDisorders_Investigation	Retinopathy Screening and photocoagulation for diabetics	No matching intervention - diagnosis is included as part of the treatment interventions	Exclude	3		Screening and diagnosis for NCD	Include	1	
CardioMetabolicDisorders_Prevention_CommunityTestingForHypertension	Community monitoring of hypertension		Exclude	3		Prevention of cardiovascular disease	Include	1	
CardioMetabolicDisorders_Prevention_WeightLoss	Prevention of cardiovascular disease		Exclude	3		Prevention of cardiovascular disease	Include	1	
CardioMetabolicDisorders_Treatment	Prevention and treatment of cardiovascular disease		Exclude	3		No matching intervention	Exclude	1	
	Hypertension		Exclude			Hypertension management and monitoring	Include		
	Diabetes Type I		NA			Diabetes type I	Include		
	Diabetes Type II		NA			Diabetes type II	Include		
	Ischemic heart disease	No CE data	NA			No matching intervention	Exclude		
	Kaposi sarcoma - first line	No CE data	NA			No matching intervention	Exclude		
	Kaposi sarcoma - second line	No CE data	NA			No matching intervention	Exclude		
	Lymphomas, nonhodgkins	No CE data	NA			No matching intervention	Exclude		
	Hodgkin's lymphoma	No CE data	NA			No matching intervention	Exclude		
AntenatalCare_FollowUp	Basic ANC		Include	1		No matching intervention	Exclude	1	
	Daily iron and folic acid supplementation (pregnant women)		Exclude			Daily iron and folic acid supplementation	Include		
AntenatalCare_Inpatient		No matching intervention	NA	2	Included since outpatient ANC in prioritised but maintained at a lower priority than PostnatalCare_Maternal_Inpatient	No matching intervention	Exclude	1	
AntenatalCare_Outpatient	Basic ANC		Include	1		No matching intervention	Exclude	1	
	Daily iron and folic acid supplementation (pregnant women)		Exclude			Daily iron and folic acid supplementation	Include		
	Syphilis detection and treatment (pregnant women)		Include			Syphilis detection and treatment	Include		
	Prenatal distribution of misoprostol (for PPH prevention)		Include			No matching intervention	Exclude		
AntenatalCare_PostAbortion	Safe abortion services		Exclude	3		No matching intervention	Exclude	1	
	Post-abortion case management		Exclude			Post-abortion case management	Include		
AntenatalCare_PostEctopicPregnancy		No matching intervention	NA	3		Ectopic case management	Include	1	
Contraception_Routine	Oral Contraception		Include	1		Oral contraception	Include	1	
	Male condom		Exclude			Male condom	Include		
	Female Condom		Exclude			No matching intervention	Exclude		
	Injectable Contraception		Include			Injectable contraception	Include		
	IUD		Include			Intrauterine device (IUD)	Include		
	Implant		Include			Implant contraception	Include		
	Tubal Ligation		Include			Tubal ligation	Include		
	Vastectomy		Include			No matching intervention	Exclude		
Copd_Treatment	COPD - Inhaled salbutamol		Exclude	3		COPD first line treatment with inhaled short acting beta agonist	Include	1	
	COPD - influenza vaccine		Exclude				NA		
	COPD - treatment of severe exacerbations		Exclude				NA		
	COPD - oxygen therapy and drugs		Exclude				NA		
Depression_TalkingTherapy	Treatment of depression		Exclude	3		Diagnosis and treatment of depression	Include	1	
	Maintenance psychosocial treatment (including antidepressants)		Exclude			Psychotherapy (PST) through problem management plus (PM+) and Friendship Bench	Include		
Depression_Treatment	Treatment of depression		Exclude	3		Diagnosis and treatment of depression	Include	1	
	Maintenance psychosocial treatment (including antidepressants)		Exclude			Psychotherapy (PST) through problem management plus (PM+) and Friendship Bench	Include		
Diarrhoea_Treatment_Inpatient	ORS and IV Fluid for severe diarrhea		Exclude	3		Acute diarrhea treatment (ORS, Zinc, IV fluids - community to secondary)	Include	1	
Diarrhoea_Treatment_Outpatient	ORS and Zinc for acute diarrhea		Exclude	3		Acute diarrhea treatment (ORS, Zinc, IV fluids - community to secondary)	Include	1	
Epi_Adolescent_Hpv	HPV vaccine		Exclude	3		HPV vaccine	Include	1	
Epi_Childhood_Bcg	BCG vaccine		Exclude	3		BCG vaccine	Include	1	
Epi_Childhood_DtpHibHep	Pentavalent (DPT-Hep-Hib)		Include	1		Pentavalent vaccine (DPT-Hep-Hib)	Include	1	
Epi_Childhood_MeaslesRubella	Measles vaccine		Include	1		Measles vaccine	Include	1	
Epi_Childhood_Opv	Polio vaccine		Exclude	3		Polio vaccine (OPV and IPV)	Include	1	
Epi_Childhood_Pneumo	Pneumococcal vaccine		Exclude	3		Pneumococcal vaccine	Include	1	
Epi_Childhood_Rota	Rotavirus vaccine		Include	1		Rotavirus vaccine	Include	1	
Epi_Pregnancy_Td	Tetanus toxoid (pregnant women)		Include	1		Tetanus toxoid	Include	1	
Epilepsy_Treatment_Followup	Anti-epileptic medication		Exclude	3		Diagnosis and treatment of epilepsy	Include	1	
Epilepsy_Treatment_Start	Anti-epileptic medication		Exclude	3		Diagnosis and treatment of epilepsy	Include	1	
FirstAttendance_NonEmergency		No matching intervention	NA	1	This is a diagnostic visit so it can't be deprioritised	NA	NA	1	This is a diagnostic visit so it can't realistically be deprioritised
Hiv_Prevention_Circumcision	Male circumcision		Include	1		No matching intervention	Exclude	3	
Hiv_Prevention_Infant	PMTCT		Include	1		Prevention of Mother to Child Transmission (PMTCT) of HIV	Include	1	
Hiv_Prevention_Prep	Pre-exposure prophylaxis for high-risk serodiscordant couples		Exclude	3		No matching intervention	Exclude	3	
	Pre-exposure prophylaxis for pregnant and breastfeeding women		Exclude			No matching intervention	Exclude		
Hiv_Test	Voluntary counselling and testing for HIV		Include	1		Voluntary counselling and testing for HIV	Include	1	
	Screen HIV+ cases for TB		Exclude			No matching intervention	Exclude		
Hiv_Treatment	ART for men		Exclude	3		Antiretroviral treatment	Include	1	
	ART for women		Exclude			Antiretroviral treatment	Include		
	Second-line ART without intensive monitoring		Exclude			Antiretroviral treatment	Include		
	Second-line ART with intensive monitoring		Exclude			Antiretroviral treatment	Include		
	Viral Load + CD4 count (Clinical monitoring and quarterly tests)		Exclude			HIV/AIDS monitoring with viral load and CD4+ count	Include		
	CD4 count (Clinical monitoring and quarterly tests)		Exclude			HIV/AIDS monitoring with viral load and CD4+ count	Include		
DeliveryCare_Basic	Vaginal delivery - skilled attendance		Include	1		Vaginal delivery (skilled attendance uncomplicated and complicated BeMONC delivery)	Include	1	
	Clean practices and immediate essential newborn care (in facility)		Include			Clean practices and immediate essential newborn care (vitamin K, erythromycin, chlorhexidine)	Include		
DeliveryCare_Comprehensive	Vaginal Delivery - with complication		Include	1		Vaginal delivery (skilled attendance uncomplicated and complicated BeMONC delivery)	Include	1	
	Cesearian section with indication		Include			Cesarian section (uncomplicated and complicated)	Include		
	Cesearian Section with indication (with complication)		Include			Cesarian section (uncomplicated and complicated)	Include		
	Management of pre-eclampsia and eclampsia		Include			Management of pre-eclampsia and eclampsia	Include		
	Management of obstructed labour		Include			Management of obstructed labour	Include		
	Active management of the 3rd stage of labour		Include			Active management of the 3rd stage of labor	Include		
	Ectopic case management		Exclude			Ectopic case management	Include		
	Antibiotics for pPRoM		Exclude			Antibiotics for preterm rupture of the membranes	Include		
	Antenatal corticosteroids for preterm labour		Include			Antenatal corticosteroids for preterm labour	Include		
			NA			Treatment of antepartum and postpartum hemorrhage	Include		
PostnatalCare_Maternal	Kangaroo mother care		Include	1		Kangaroo mother care	Include	1	
	Support for breastfeeding mothers		Include			Support of breastfeeding mothers	Include		
PostnatalCare_Maternal_Inpatient	Maternal sepsis case management		Include	1		Maternal sepsis case management	Include	1	
Malaria_Prevention_Iptp	Intermittent preventive treatment in infants (IPTi)		Include	1		No matching intervention	Exclude	3	
	IPT (pregnant women)		Exclude			IPT distribution	Include		
Malaria_Test	No matching intervention	No matching intervention - included in treatment	NA	1	Prioritised despite no direct match with LCOA interventions because it covered in the treatment intervention	Rapid diagnosis tests	Include	1	
Malaria_Treatment_Complicated	No matching intervention	No matching intervention	NA	1	Included since uncomplicated malaria treatment is prioritised	Complicated malaria treatment	Include	1	
	Complicated (children, injectable artesunate)		Include			Complicated malaria treatment	Include		
Malaria_Treatment	Uncomplicated malaria treatment		Include	1		Uncomplicated first line malaria treatment including rectal treatment for <5 years old and home treatment	Include	1	
	Home management of fevers using antimalarial (artesunate-amodiaquine AAQ)		Exclude			Uncomplicated first line malaria treatment including rectal treatment for <5 years old and home treatment	Include		
	Home management of fevers using antimalarial (artesunate-amodiaquine AAQ) (under 5)		Exclude			Uncomplicated first line malaria treatment including rectal treatment for <5 years old and home treatment	Include		
	First line malaria treatment with Dihydroartemisinin piperaquin (Children)		Include			Uncomplicated first line malaria treatment including rectal treatment for <5 years old and home treatment	Include		
	Home management of fevers using antimalarial (artesunate-amodiaquine AAQ)		Exclude			Uncomplicated first line malaria treatment including rectal treatment for <5 years old and home treatment	Include		
Measles_Treatment	Child Health Weeks (CHWs)- semi annual, campaign-style, facility- and outreach-based events for provision of measles vaccination, vitamin A and deworming	Unclear CE data	NA	2	Included with low priority since no-matching intervention was found	No matching intervention	Exclude	3	
DeliveryCare_Neonatal	Neonatal resuscitation (institutional)		Include	1		Neonatal resuscitation	Include	1	
PostnatalCare_Neonatal	Clean practices and immediate essential newborn care (in facility)		Include	1		Clean practices and immediate essential newborn care (vitamin K, erythromycin, chlorhexidine)	Include	1	
PostnatalCare_Neonatal_Inpatient	Newborn sepsis - full supportive care		Include	1		Essential care of preterm of sick newborn including sepsis management	Include	1	
OesophagealCancer_Investigation	Head and neck cancer (esophageal)		NA	3	Excluded because tertiary services/ cancer are generally excluded under LCOA	No matching intervention	Exclude	3	
OesophagealCancer_PalliativeCare	Head and neck cancer (esophageal)	No CE data	NA	3	Excluded because tertiary services/ cancer are generally excluded under LCOA	No matching intervention	Exclude	3	
OesophagealCancer_Treatment	Head and neck cancer (esophageal)	No CE data	NA	3	Excluded because tertiary services/ cancer are generally excluded under LCOA	No matching intervention	Exclude	3	
OtherAdultCancer_Investigation	Colorectoral cancer (screening + treatment)		Exclude	3		No matching intervention	Exclude	3	
	Cervical cancer screening		Exclude			Cervical cancer screening	Include		
OtherAdultCancer_PalliativeCare		No matching intervention	NA	3	Excluded because tertiary services/ cancer are generally excluded under LCOA	No matching intervention	Exclude	3	
OtherAdultCancer_Treatment	Cervical cancer (first line)		Exclude	3		Treatment of precancerous lesions	Include	2	Assigned 2 because only one type of "Other Adult Cancer" is included
	GIT, intestine cancer		Exclude			No matching intervention	Exclude		
	Colorectoral cancer (treatment)		Exclude			No matching intervention	Exclude		
PostnatalCare_TreatmentForObstetricFistula	Fistula repair surgery		Include	1		Fistula repair	Include	1	
ProstateCancer_Investigation		No matching intervention	NA	3	Excluded because tertiary services/ cancer are generally excluded under LCOA	No matching intervention	Exclude	3	
ProstateCancer_PalliativeCare		No matching intervention	NA	3	Excluded because tertiary services/ cancer are generally excluded under LCOA	No matching intervention	Exclude	3	
ProstateCancer_Treatment		No matching intervention	NA	3	Excluded because tertiary services/ cancer are generally excluded under LCOA	No matching intervention	Exclude	3	
Rti_AcutePainManagement		No matching intervention	NA	2	Included since no matching intervention	No matching intervention	Exclude	1	it is assumed that this is part of treatment of injuries in the EHP
Rti_BurnManagement		No matching intervention	NA	2	Not excluded because no match	No matching intervention	Exclude	3	
Rti_FractureCast	Treatment of injuries (Fracture reduction)		Exclude	3		Treatment of injuries – fracture reduction & fixation	Include	1	
Rti_Imaging		No matching intervention	NA	2	Diagnostic	No matching intervention	Exclude	1	it is assumed that this is part of treatment of injuries in the EHP
Rti_MajorSurgeries		No matching intervention	NA	3	Excluded because terteiary services are generally excluded under LCOA	No matching intervention	Exclude	1	it is assumed that this is part of treatment of injuries in the EHP
Rti_MedicalIntervention		No matching intervention	NA	2	Not excluded because no match	No matching intervention	Exclude	1	it is assumed that this is part of treatment of injuries in the EHP
Rti_MinorSurgeries		No matching intervention	NA	2	Not excluded because no match	Treatment of injuries – blunt and soft tissue	Include	1	
Rti_OpenFractureTreatment	Treatment of injuries (Fracture fixation)		Exclude	3		Treatment of injuries – fracture reduction & fixation	Include	1	
Rti_ShockTreatment		No matching intervention	NA	2	Not excluded because no match	No matching intervention	Exclude	1	it is assumed that this is part of treatment of injuries in the EHP
Rti_Suture		No matching intervention	NA	2	Not excluded because no match	No matching intervention	Exclude	1	it is assumed that this is part of treatment of injuries in the EHP
Rti_TetanusVaccine		No matching intervention	NA	2	Not excluded because no match	No matching intervention	Exclude	1	it is assumed that this is part of treatment of injuries in the EHP
Schisto_MDA	Schistosomiasis mass drug administration (adults)	No matching intervention	Exclude	3		No matching intervention	Exclude	3	
	Schistosomiasis mass drug administration (school children)		Include			No matching intervention	Exclude		
Schisto_Treatment	Schistosomiasis treatment	No CE data	NA	2	Not excluded because no match	Schistosomiasis diagnosis (stool and urine)	Include	1	
						Schistosomiasis treatment	Include		
Undernutrition_Feeding	Community-based management of moderate acute malnutrition (children)		Exclude	1		Community-based management of moderate malnutrition	Include	1	
	Management of severe malnutrition (children)		Exclude			Community-based management of severe malnutrition	Include		
	Management of severe malnutrition (children)					NRU management of severe complicated malnutrition	Include		
	Provision of supplementary food and nutrition counselling with growth monitoring		Exclude			Growth monitoring the promotion	Include		
	Vitamin A supplementation in infants and children 6-59 months		Exclude			Vitamin A supplementation and deworming in infants and children 6-59 months	Include		
	Management of moderate acute malnutrition (children) with ready-to-use supplementary foods (RUSF)		Include			No matching intervention	Exclude		
Tb_Prevention_Ipt	Isonized Preventive Therapy for HIV+ people		Include	1		No matching intervention	Exclude	1	
	Isonized Preventive Therapy for HIV+ people		Include			3HP preventative therapy for PLHIV	Include		
	Isonized Preventive Therapy for children in contact with TB patients		Exclude			Isoniazid preventative therapy for children in contact with TB cases	Include		
	Isonized Preventive Therapy for HIV+ pregnant women		Include			Isoniazid preventative therapy for HIV+ pregnant women	Include		
Tb_Test_FollowUp		No matching intervention	NA	2	Included (but not high priority) because some kind of follow-up is required to deliver Tb_Treatment (only Xpert is excluded based on LCOA)	No matching intervention	Exclude	1	
Tb_Test_Screening	Targeted Xpert for patients with presumptive tuberculosis (Smear negative, HIV positive, retreatment, contacts of MDR-TB cases)		Exclude	1	Prioritised because some kind of screening is required to deliver Tb_Treatment (only Xpert is excluded based on LCOA)	GeneXpert diagnosis for all patients with presumptive TB	Include	1	
	(Full) Xpert for all patients with presumptive tuberculosis		Exclude			No matching intervention	Exclude		
Tb_Test_Xray	Chest X-ray	No CE data	NA	1	Prioritised because some kind of screening is required to deliver Tb_Treatment	No matching intervention	Exclude	1	
Tb_Treatment	First line treatment of smear positive cases (95% coverage)		Include	1		First line treatment for TB with DOTS for high-risk patients	Include	1	
	Full DOTS (smear-positive, smear negative and Extrapulmonary cases)		Exclude			MDR-TB treatment and case management	Include		
**Lowest priority level which is included for delivery in the policy**	2					2			
