# Supplementary material for: A new approach to Health Benefits Package design: an application of the Thanzi La Onse model in Malawi
Source: PLoS Comput Biol. 2024 Sep 30;20(9):e1012462. doi: 10.1371/journal.pcbi.1012462 (PMC11567512; doi:10.1371/journal.pcbi.1012462)
Supplement: S3 Appendix — (DOCX) [file pcbi.1012462.s003.docx]

**Motivation of individual prioritisation policies**

In this section we outline the prioritisation policies that will be evaluated in this work, as well as their individual motivations. We emphasise again that the prioritisation of children with emergencies followed by adults with emergencies above all other treatments is adopted a priori by all policies evaluated. A detailed breakdown of the priority associated with each treatment type and vulnerable category is also provided. Notice that the lower the priority value, the higher the prioritisation (i.e. priority “0” is the highest possible prioritisation a treatment can have, and treatments with priority “1”, “2”, “3”, ... have a progressively lower prioritisation).

Each table includes:

- The priority assigned by default, under that policy, to each of the treatments modelled in the TLO simulation;
- The priority assigned to the same treatment, under that policy. If the patient falls into one of the eligible vulnerable categories (five years old or under, pregnant, diagnosed with TB, or diagnosed with HIV). If no such value is specified, patients belonging to those vulnerable categories are assigned a default priority.
- The lowest prioritisation (or highest priority) considered under that policy. Any treatment assigned a lower prioritisation (and hence higher priority value) is completely excluded from the healthcare system provision.

***“No policy” (NP) Policy***

Under this policy, all healthcare system interactions are assigned equal priority, and are therefore delivered in a completely random order until capabilities for the day are exhausted. This mimics a scenario where, aside from emergency procedures, no preference in the kind of services that the healthcare system should deliver while operating under strict resource constraints is enforced. This policy is the opposite of an HBP-approach, and will therefore provide a benchmark against which all others can be evaluated, to establish whether introducing the prioritisation they espouse can lead to an overall improvement in health outcomes without expanding existing resource capacities.

| **Treatment** | **Priority** | **If 5 or Under** | **If pregnant** | **If TB diagnosed** | **If HIV diagnosed** |
| --- | --- | --- | --- | --- | --- |
| **FirstAttendance_Emergency** | 1 | 0 | -1 | -1 | -1 |
| **FirstAttendance_SpuriousEmergencyCare** | 1 | 0 | -1 | -1 | -1 |
| **Alri_Pneumonia_Treatment_Outpatient** | 2 | -1 | -1 | -1 | -1 |
| **Alri_Pneumonia_Treatment_Inpatient** | 2 | -1 | -1 | -1 | -1 |
| **Alri_Pneumonia_Treatment_Inpatient_Followup** | 2 | -1 | -1 | -1 | -1 |
| **BladderCancer_Investigation** | 2 | -1 | -1 | -1 | -1 |
| **BladderCancer_PalliativeCare** | 2 | -1 | -1 | -1 | -1 |
| **BladderCancer_Treatment** | 2 | -1 | -1 | -1 | -1 |
| **BreastCancer_Investigation** | 2 | -1 | -1 | -1 | -1 |
| **BreastCancer_PalliativeCare** | 2 | -1 | -1 | -1 | -1 |
| **BreastCancer_Treatment** | 2 | -1 | -1 | -1 | -1 |
| **CardioMetabolicDisorders_Investigation** | 2 | -1 | -1 | -1 | -1 |
| **CardioMetabolicDisorders_Prevention_CommunityTestingForHypertension** | 2 | -1 | -1 | -1 | -1 |
| **CardioMetabolicDisorders_Prevention_WeightLoss** | 2 | -1 | -1 | -1 | -1 |
| **CardioMetabolicDisorders_Treatment** | 2 | -1 | -1 | -1 | -1 |
| **AntenatalCare_FollowUp** | 2 | -1 | -1 | -1 | -1 |
| **AntenatalCare_Inpatient** | 2 | -1 | -1 | -1 | -1 |
| **AntenatalCare_Outpatient** | 2 | -1 | -1 | -1 | -1 |
| **AntenatalCare_PostAbortion** | 2 | -1 | -1 | -1 | -1 |
| **AntenatalCare_PostEctopicPregnancy** | 2 | -1 | -1 | -1 | -1 |
| **Contraception_Routine** | 2 | -1 | -1 | -1 | -1 |
| **Copd_Treatment** | 2 | -1 | -1 | -1 | -1 |
| **Depression_TalkingTherapy** | 2 | -1 | -1 | -1 | -1 |
| **Depression_Treatment** | 2 | -1 | -1 | -1 | -1 |
| **Diarrhoea_Treatment_Inpatient** | 2 | -1 | -1 | -1 | -1 |
| **Diarrhoea_Treatment_Outpatient** | 2 | -1 | -1 | -1 | -1 |
| **Epi_Adolescent_Hpv** | 2 | -1 | -1 | -1 | -1 |
| **Epi_Childhood_Bcg** | 2 | -1 | -1 | -1 | -1 |
| **Epi_Childhood_DtpHibHep** | 2 | -1 | -1 | -1 | -1 |
| **Epi_Childhood_MeaslesRubella** | 2 | -1 | -1 | -1 | -1 |
| **Epi_Childhood_Opv** | 2 | -1 | -1 | -1 | -1 |
| **Epi_Childhood_Pneumo** | 2 | -1 | -1 | -1 | -1 |
| **Epi_Childhood_Rota** | 2 | -1 | -1 | -1 | -1 |
| **Epi_Pregnancy_Td** | 2 | -1 | -1 | -1 | -1 |
| **Epilepsy_Treatment_Followup** | 2 | -1 | -1 | -1 | -1 |
| **Epilepsy_Treatment_Start** | 2 | -1 | -1 | -1 | -1 |
| **FirstAttendance_NonEmergency** | 2 | -1 | -1 | -1 | -1 |
| **Hiv_Prevention_Circumcision** | 2 | -1 | -1 | -1 | -1 |
| **Hiv_Prevention_Infant** | 2 | -1 | -1 | -1 | -1 |
| **Hiv_Prevention_Prep** | 2 | -1 | -1 | -1 | -1 |
| **Hiv_Test** | 2 | -1 | -1 | -1 | -1 |
| **Hiv_Treatment** | 2 | -1 | -1 | -1 | -1 |
| **DeliveryCare_Basic** | 2 | -1 | -1 | -1 | -1 |
| **DeliveryCare_Comprehensive** | 2 | -1 | -1 | -1 | -1 |
| **PostnatalCare_Maternal** | 2 | -1 | -1 | -1 | -1 |
| **PostnatalCare_Maternal_Inpatient** | 2 | -1 | -1 | -1 | -1 |
| **Malaria_Prevention_Iptp** | 2 | -1 | -1 | -1 | -1 |
| **Malaria_Test** | 2 | -1 | -1 | -1 | -1 |
| **Malaria_Treatment** | 2 | -1 | -1 | -1 | -1 |
| **Malaria_Treatment_Complicated** | 2 | -1 | -1 | -1 | -1 |
| **Measles_Treatment** | 2 | -1 | -1 | -1 | -1 |
| **PostnatalCare_Neonatal** | 2 | -1 | -1 | -1 | -1 |
| **PostnatalCare_Neonatal_Inpatient** | 2 | -1 | -1 | -1 | -1 |
| **OesophagealCancer_Investigation** | 2 | -1 | -1 | -1 | -1 |
| **OesophagealCancer_PalliativeCare** | 2 | -1 | -1 | -1 | -1 |
| **OesophagealCancer_Treatment** | 2 | -1 | -1 | -1 | -1 |
| **OtherAdultCancer_Investigation** | 2 | -1 | -1 | -1 | -1 |
| **OtherAdultCancer_PalliativeCare** | 2 | -1 | -1 | -1 | -1 |
| **OtherAdultCancer_Treatment** | 2 | -1 | -1 | -1 | -1 |
| **PostnatalCare_TreatmentForObstetricFistula** | 2 | -1 | -1 | -1 | -1 |
| **ProstateCancer_Investigation** | 2 | -1 | -1 | -1 | -1 |
| **ProstateCancer_PalliativeCare** | 2 | -1 | -1 | -1 | -1 |
| **ProstateCancer_Treatment** | 2 | -1 | -1 | -1 | -1 |
| **Rti_AcutePainManagement** | 2 | -1 | -1 | -1 | -1 |
| **Rti_BurnManagement** | 2 | -1 | -1 | -1 | -1 |
| **Rti_FractureCast** | 2 | -1 | -1 | -1 | -1 |
| **Rti_Imaging** | 2 | -1 | -1 | -1 | -1 |
| **Rti_MajorSurgeries** | 2 | -1 | -1 | -1 | -1 |
| **Rti_MedicalIntervention** | 2 | -1 | -1 | -1 | -1 |
| **Rti_MinorSurgeries** | 2 | -1 | -1 | -1 | -1 |
| **Rti_OpenFractureTreatment** | 2 | -1 | -1 | -1 | -1 |
| **Rti_ShockTreatment** | 2 | -1 | -1 | -1 | -1 |
| **Rti_Suture** | 2 | -1 | -1 | -1 | -1 |
| **Rti_TetanusVaccine** | 2 | -1 | -1 | -1 | -1 |
| **Schisto_MDA** | 2 | -1 | -1 | -1 | -1 |
| **Schisto_Treatment** | 2 | -1 | -1 | -1 | -1 |
| **Undernutrition_Feeding** | 2 | -1 | -1 | -1 | -1 |
| **Tb_Prevention_Ipt** | 2 | -1 | -1 | -1 | -1 |
| **Tb_Test_Clinical** | 2 | -1 | -1 | -1 | -1 |
| **Tb_Test_FollowUp** | 2 | -1 | -1 | -1 | -1 |
| **Tb_Test_Screening** | 2 | -1 | -1 | -1 | -1 |
| **Tb_Test_Xray** | 2 | -1 | -1 | -1 | -1 |
| **Tb_Treatment** | 2 | -1 | -1 | -1 | -1 |
| **Lowest priority considered** | 2 |  |  |  |  |

***Linear Constrained Optimisation Analysis (Naive LCOA)***

This policy is based on the output of the linear constrained optimisation analysis set up to maximise the net health benefit from interventions given Malawi’s health system resource constraints (drug budget and size of the health workforce), and demand constraints (maximum feasible coverage of each intervention given demand/access constraints), assuming a cost-effectiveness threshold/health opportunity cost of $65.8/DALY averted [1, 2]. For our paper, we consider the LCOA output which does not consider donor constraints and therefore “naively” maximises net health benefit given the cost-effectiveness evidence available in 2021 and assuming perfect fungibility of the health sector budget across disease programs. For a more detailed description of the methodology and results, see [1].

Because this approach relied on cost-effectiveness evidence on individual interventions available in the current literature, it was blind to interventions on which this evidence was not available. As stated in [2], “in the absence of adequate evidence, the inclusion of other interventions should be based on expert opinion and deliberation, followed by “squeezing out” interventions from the theoretical optimal HBP to account for resources committed to these additional interventions”. In order to accommodate this recommendation, instead of excluding treatment IDs which were not considered under LCOA from this policy, we include them with a lower priority level than those which were included in the prioritised list of interventions proposed by the LCOA. Under this policy, other interventions which were included in the analysis but excluded from the final list of prioritised interventions will, unlike in the case of ’No Policy’, be strictly excluded from the health care provision.

Below, we provide a breakdown of the priority level associated with each treatment as well as the mapping between interventions considered by the LCOA approach and the treatment IDs modelled by the TLO simulation.

| **Treatment** | **Priority** | **If 5 or under** | **If pregnant** | **If TB diagnosed** | **FT_if_Hivdiagnosed** |
| --- | --- | --- | --- | --- | --- |
| **FirstAttendance_Emergency** | 1 | 0 | -1 | -1 | -1 |
| **FirstAttendance_SpuriousEmergencyCare** | 1 | 0 | -1 | -1 | -1 |
| **Alri_Pneumonia_Treatment_Outpatient** | 2 | -1 | -1 | -1 | -1 |
| **Alri_Pneumonia_Treatment_Inpatient** | 3 | -1 | -1 | -1 | -1 |
| **Alri_Pneumonia_Treatment_Inpatient_Followup** | 3 | -1 | -1 | -1 | -1 |
| **BladderCancer_Investigation** | 4 | -1 | -1 | -1 | -1 |
| **BladderCancer_PalliativeCare** | 4 | -1 | -1 | -1 | -1 |
| **BladderCancer_Treatment** | 4 | -1 | -1 | -1 | -1 |
| **BreastCancer_Investigation** | 4 | -1 | -1 | -1 | -1 |
| **BreastCancer_PalliativeCare** | 4 | -1 | -1 | -1 | -1 |
| **BreastCancer_Treatment** | 4 | -1 | -1 | -1 | -1 |
| **CardioMetabolicDisorders_Investigation** | 4 | -1 | -1 | -1 | -1 |
| **CardioMetabolicDisorders_Prevention_CommunityTestingForHypertension** | 4 | -1 | -1 | -1 | -1 |
| **CardioMetabolicDisorders_Prevention_WeightLoss** | 4 | -1 | -1 | -1 | -1 |
| **CardioMetabolicDisorders_Treatment** | 4 | -1 | -1 | -1 | -1 |
| **AntenatalCare_FollowUp** | 2 | -1 | -1 | -1 | -1 |
| **AntenatalCare_Inpatient** | 3 | -1 | -1 | -1 | -1 |
| **AntenatalCare_Outpatient** | 2 | -1 | -1 | -1 | -1 |
| **AntenatalCare_PostAbortion** | 4 | -1 | -1 | -1 | -1 |
| **AntenatalCare_PostEctopicPregnancy** | 4 | -1 | -1 | -1 | -1 |
| **Contraception_Routine** | 2 | -1 | -1 | -1 | -1 |
| **Copd_Treatment** | 4 | -1 | -1 | -1 | -1 |
| **Depression_TalkingTherapy** | 4 | -1 | -1 | -1 | -1 |
| **Depression_Treatment** | 4 | -1 | -1 | -1 | -1 |
| **Diarrhoea_Treatment_Inpatient** | 4 | -1 | -1 | -1 | -1 |
| **Diarrhoea_Treatment_Outpatient** | 4 | -1 | -1 | -1 | -1 |
| **Epi_Adolescent_Hpv** | 4 | -1 | -1 | -1 | -1 |
| **Epi_Childhood_Bcg** | 4 | -1 | -1 | -1 | -1 |
| **Epi_Childhood_DtpHibHep** | 2 | -1 | -1 | -1 | -1 |
| **Epi_Childhood_MeaslesRubella** | 2 | -1 | -1 | -1 | -1 |
| **Epi_Childhood_Opv** | 4 | -1 | -1 | -1 | -1 |
| **Epi_Childhood_Pneumo** | 4 | -1 | -1 | -1 | -1 |
| **Epi_Childhood_Rota** | 2 | -1 | -1 | -1 | -1 |
| **Epi_Pregnancy_Td** | 2 | -1 | -1 | -1 | -1 |
| **Epilepsy_Treatment_Followup** | 4 | -1 | -1 | -1 | -1 |
| **Epilepsy_Treatment_Start** | 4 | -1 | -1 | -1 | -1 |
| **FirstAttendance_NonEmergency** | 2 | -1 | -1 | -1 | -1 |
| **Hiv_Prevention_Circumcision** | 2 | -1 | -1 | -1 | -1 |
| **Hiv_Prevention_Infant** | 2 | -1 | -1 | -1 | -1 |
| **Hiv_Prevention_Prep** | 4 | -1 | -1 | -1 | -1 |
| **Hiv_Test** | 2 | -1 | -1 | -1 | -1 |
| **Hiv_Treatment** | 4 | -1 | -1 | -1 | -1 |
| **DeliveryCare_Basic** | 2 | -1 | -1 | -1 | -1 |
| **DeliveryCare_Comprehensive** | 2 | -1 | -1 | -1 | -1 |
| **PostnatalCare_Maternal** | 2 | -1 | -1 | -1 | -1 |
| **PostnatalCare_Maternal_Inpatient** | 2 | -1 | -1 | -1 | -1 |
| **Malaria_Prevention_Iptp** | 2 | -1 | -1 | -1 | -1 |
| **Malaria_Test** | 2 | -1 | -1 | -1 | -1 |
| **Malaria_Treatment** | 2 | -1 | -1 | -1 | -1 |
| **Malaria_Treatment_Complicated** | 2 | -1 | -1 | -1 | -1 |
| **Measles_Treatment** | 3 | -1 | -1 | -1 | -1 |
| **PostnatalCare_Neonatal** | 2 | -1 | -1 | -1 | -1 |
| **PostnatalCare_Neonatal_Inpatient** | 2 | -1 | -1 | -1 | -1 |
| **OesophagealCancer_Investigation** | 4 | -1 | -1 | -1 | -1 |
| **OesophagealCancer_PalliativeCare** | 4 | -1 | -1 | -1 | -1 |
| **OesophagealCancer_Treatment** | 4 | -1 | -1 | -1 | -1 |
| **OtherAdultCancer_Investigation** | 4 | -1 | -1 | -1 | -1 |
| **OtherAdultCancer_PalliativeCare** | 4 | -1 | -1 | -1 | -1 |
| **OtherAdultCancer_Treatment** | 4 | -1 | -1 | -1 | -1 |
| **PostnatalCare_TreatmentForObstetricFistula** | 2 | -1 | -1 | -1 | -1 |
| **ProstateCancer_Investigation** | 4 | -1 | -1 | -1 | -1 |
| **ProstateCancer_PalliativeCare** | 4 | -1 | -1 | -1 | -1 |
| **ProstateCancer_Treatment** | 4 | -1 | -1 | -1 | -1 |
| **Rti_AcutePainManagement** | 3 | -1 | -1 | -1 | -1 |
| **Rti_BurnManagement** | 3 | -1 | -1 | -1 | -1 |
| **Rti_FractureCast** | 4 | -1 | -1 | -1 | -1 |
| **Rti_Imaging** | 3 | -1 | -1 | -1 | -1 |
| **Rti_MajorSurgeries** | 4 | -1 | -1 | -1 | -1 |
| **Rti_MedicalIntervention** | 3 | -1 | -1 | -1 | -1 |
| **Rti_MinorSurgeries** | 3 | -1 | -1 | -1 | -1 |
| **Rti_OpenFractureTreatment** | 4 | -1 | -1 | -1 | -1 |
| **Rti_ShockTreatment** | 3 | -1 | -1 | -1 | -1 |
| **Rti_Suture** | 3 | -1 | -1 | -1 | -1 |
| **Rti_TetanusVaccine** | 3 | -1 | -1 | -1 | -1 |
| **Schisto_MDA** | 4 | -1 | -1 | -1 | -1 |
| **Schisto_Treatment** | 3 | -1 | -1 | -1 | -1 |
| **Undernutrition_Feeding** | 2 | -1 | -1 | -1 | -1 |
| **Tb_Prevention_Ipt** | 2 | -1 | -1 | -1 | -1 |
| **Tb_Test_Clinical** | 2 | -1 | -1 | -1 | -1 |
| **Tb_Test_FollowUp** | 3 | -1 | -1 | -1 | -1 |
| **Tb_Test_Screening** | 2 | -1 | -1 | -1 | -1 |
| **Tb_Test_Xray** | 2 | -1 | -1 | -1 | -1 |
| **Tb_Treatment** | 2 | -1 | -1 | -1 | -1 |
| **Lowest priority considered** | 3 |  |  |  |  |

***HBP from Health Sector Strategic Plan III (HSSP-III HBP)***

This policy is based on the HBP adopted in the Health Sector Strategic Plan III [3], the Government of Malawi’s latest medium-term strategic plan for the health sector. The HSSP-III HBP was developed using a combination of two methods – LCOA (described above) and multi- criteria decision analysis (MCDA). The MCDA involved a group of programmatic, clinical, and policy experts assigning scores to candidate interventions for inclusion into the HBP based on the following five criteria - severity, effectiveness, poverty reduction, vulnerable populations, and level of care - in alignment with HSSP-III values [1]. Following the MCDA score assignment, a further deliberative process was conducted to make a final decision on the inclusion and exclusion of interventions based on the combined results of the LCOA and MCDA processes. For a more detailed description of the methodology and results, see [1].

As under the Naive LCOA approach, this policy will strictly exclude interventions not prioritised by the HSSP-III from the health care provision. In the supplementary material, we provide a breakdown of the priority level associated with each treatment as well as the mapping between interventions considered in the HBP design process and the treatment IDs modelled by the TLO simulation.

| **Treatment** | **Priority** | **If 5 years or younger** | **If pregnant** | **If TB diagnosed** | **If HIV diagnosed** |
| --- | --- | --- | --- | --- | --- |
| **FirstAttendance_Emergency** | 1 | 0 | -1 | -1 | -1 |
| **FirstAttendance_SpuriousEmergencyCare** | 1 | 0 | -1 | -1 | -1 |
| **Alri_Pneumonia_Treatment_Outpatient** | 2 | -1 | -1 | -1 | -1 |
| **Alri_Pneumonia_Treatment_Inpatient** | 2 | -1 | -1 | -1 | -1 |
| **Alri_Pneumonia_Treatment_Inpatient_Followup** | 2 | -1 | -1 | -1 | -1 |
| **BladderCancer_Investigation** | 4 | -1 | -1 | -1 | -1 |
| **BladderCancer_PalliativeCare** | 4 | -1 | -1 | -1 | -1 |
| **BladderCancer_Treatment** | 4 | -1 | -1 | -1 | -1 |
| **BreastCancer_Investigation** | 4 | -1 | -1 | -1 | -1 |
| **BreastCancer_PalliativeCare** | 4 | -1 | -1 | -1 | -1 |
| **BreastCancer_Treatment** | 4 | -1 | -1 | -1 | -1 |
| **CardioMetabolicDisorders_Investigation** | 2 | -1 | -1 | -1 | -1 |
| **CardioMetabolicDisorders_Prevention_CommunityTestingForHypertension** | 2 | -1 | -1 | -1 | -1 |
| **CardioMetabolicDisorders_Prevention_WeightLoss** | 2 | -1 | -1 | -1 | -1 |
| **CardioMetabolicDisorders_Treatment** | 2 | -1 | -1 | -1 | -1 |
| **AntenatalCare_FollowUp** | 2 | -1 | -1 | -1 | -1 |
| **AntenatalCare_Inpatient** | 2 | -1 | -1 | -1 | -1 |
| **AntenatalCare_Outpatient** | 2 | -1 | -1 | -1 | -1 |
| **AntenatalCare_PostAbortion** | 2 | -1 | -1 | -1 | -1 |
| **AntenatalCare_PostEctopicPregnancy** | 2 | -1 | -1 | -1 | -1 |
| **Contraception_Routine** | 2 | -1 | -1 | -1 | -1 |
| **Copd_Treatment** | 2 | -1 | -1 | -1 | -1 |
| **Depression_TalkingTherapy** | 2 | -1 | -1 | -1 | -1 |
| **Depression_Treatment** | 2 | -1 | -1 | -1 | -1 |
| **Diarrhoea_Treatment_Inpatient** | 2 | -1 | -1 | -1 | -1 |
| **Diarrhoea_Treatment_Outpatient** | 2 | -1 | -1 | -1 | -1 |
| **Epi_Adolescent_Hpv** | 2 | -1 | -1 | -1 | -1 |
| **Epi_Childhood_Bcg** | 2 | -1 | -1 | -1 | -1 |
| **Epi_Childhood_DtpHibHep** | 2 | -1 | -1 | -1 | -1 |
| **Epi_Childhood_MeaslesRubella** | 2 | -1 | -1 | -1 | -1 |
| **Epi_Childhood_Opv** | 2 | -1 | -1 | -1 | -1 |
| **Epi_Childhood_Pneumo** | 2 | -1 | -1 | -1 | -1 |
| **Epi_Childhood_Rota** | 2 | -1 | -1 | -1 | -1 |
| **Epi_Pregnancy_Td** | 2 | -1 | -1 | -1 | -1 |
| **Epilepsy_Treatment_Followup** | 2 | -1 | -1 | -1 | -1 |
| **Epilepsy_Treatment_Start** | 2 | -1 | -1 | -1 | -1 |
| **FirstAttendance_NonEmergency** | 2 | -1 | -1 | -1 | -1 |
| **Hiv_Prevention_Circumcision** | 4 | -1 | -1 | -1 | -1 |
| **Hiv_Prevention_Infant** | 2 | -1 | -1 | -1 | -1 |
| **Hiv_Prevention_Prep** | 4 | -1 | -1 | -1 | -1 |
| **Hiv_Test** | 2 | -1 | -1 | -1 | -1 |
| **Hiv_Treatment** | 2 | -1 | -1 | -1 | -1 |
| **DeliveryCare_Basic** | 2 | -1 | -1 | -1 | -1 |
| **DeliveryCare_Comprehensive** | 2 | -1 | -1 | -1 | -1 |
| **PostnatalCare_Maternal** | 2 | -1 | -1 | -1 | -1 |
| **PostnatalCare_Maternal_Inpatient** | 2 | -1 | -1 | -1 | -1 |
| **Malaria_Prevention_Iptp** | 4 | -1 | -1 | -1 | -1 |
| **Malaria_Test** | 2 | -1 | -1 | -1 | -1 |
| **Malaria_Treatment** | 2 | -1 | -1 | -1 | -1 |
| **Malaria_Treatment_Complicated** | 2 | -1 | -1 | -1 | -1 |
| **Measles_Treatment** | 4 | -1 | -1 | -1 | -1 |
| **PostnatalCare_Neonatal** | 2 | -1 | -1 | -1 | -1 |
| **PostnatalCare_Neonatal_Inpatient** | 2 | -1 | -1 | -1 | -1 |
| **OesophagealCancer_Investigation** | 4 | -1 | -1 | -1 | -1 |
| **OesophagealCancer_PalliativeCare** | 4 | -1 | -1 | -1 | -1 |
| **OesophagealCancer_Treatment** | 4 | -1 | -1 | -1 | -1 |
| **OtherAdultCancer_Investigation** | 4 | -1 | -1 | -1 | -1 |
| **OtherAdultCancer_PalliativeCare** | 4 | -1 | -1 | -1 | -1 |
| **OtherAdultCancer_Treatment** | 3 | -1 | -1 | -1 | -1 |
| **PostnatalCare_TreatmentForObstetricFistula** | 2 | -1 | -1 | -1 | -1 |
| **ProstateCancer_Investigation** | 4 | -1 | -1 | -1 | -1 |
| **ProstateCancer_PalliativeCare** | 4 | -1 | -1 | -1 | -1 |
| **ProstateCancer_Treatment** | 4 | -1 | -1 | -1 | -1 |
| **Rti_AcutePainManagement** | 2 | -1 | -1 | -1 | -1 |
| **Rti_BurnManagement** | 4 | -1 | -1 | -1 | -1 |
| **Rti_FractureCast** | 2 | -1 | -1 | -1 | -1 |
| **Rti_Imaging** | 2 | -1 | -1 | -1 | -1 |
| **Rti_MajorSurgeries** | 2 | -1 | -1 | -1 | -1 |
| **Rti_MedicalIntervention** | 2 | -1 | -1 | -1 | -1 |
| **Rti_MinorSurgeries** | 2 | -1 | -1 | -1 | -1 |
| **Rti_OpenFractureTreatment** | 2 | -1 | -1 | -1 | -1 |
| **Rti_ShockTreatment** | 2 | -1 | -1 | -1 | -1 |
| **Rti_Suture** | 2 | -1 | -1 | -1 | -1 |
| **Rti_TetanusVaccine** | 2 | -1 | -1 | -1 | -1 |
| **Schisto_MDA** | 4 | -1 | -1 | -1 | -1 |
| **Schisto_Treatment** | 2 | -1 | -1 | -1 | -1 |
| **Undernutrition_Feeding** | 2 | -1 | -1 | -1 | -1 |
| **Tb_Prevention_Ipt** | 2 | -1 | -1 | -1 | -1 |
| **Tb_Test_Clinical** | 2 | -1 | -1 | -1 | -1 |
| **Tb_Test_FollowUp** | 2 | -1 | -1 | -1 | -1 |
| **Tb_Test_Screening** | 2 | -1 | -1 | -1 | -1 |
| **Tb_Test_Xray** | 2 | -1 | -1 | -1 | -1 |
| **Tb_Treatment** | 2 | -1 | -1 | -1 | -1 |
| **Lowest priority considered** | 3 |  |  |  |  |

***“Vertical Programmes” (VP) Policy***

The public health sector in Malawi is heavily reliant on the support of international donors, estimated to contribute 70% of its overall budget [4, 5]. Most of this support takes the form of vertical programmes, which channel financial resources directly towards areas of health and services that are the focus of the funding agency, rather than to the public healthcare system as a whole. So far, this kind of direct funding has primarily benefited malaria, HIV/AIDS, TB, and immunisation programmes [6]. The extent to which this lack of integration is beneficial or detrimental to the achievement of health targets, and to the overall functioning and strengthening of the public healthcare system in general, is difficult to establish [6]. A simpler question that can be asked, however, is whether an allocation of resources driven by funding agencies’ preferences rather than a holistic assessment of health needs may be limiting the health outcome that could be achieved if those same resources were redistributed across a much larger range of services [7]. This is the question we seek to address via the evaluation of the VP policy, in which all services related to malaria, HIV/AIDS, TB, and immunisation are prioritised over all other interventions.

| **Treatment** | **Priority** | **If 5 years or under** | **If pregnant** | **If TB diagnosed** | **If HIV diagnosed** |
| --- | --- | --- | --- | --- | --- |
| **FirstAttendance_Emergency** | 1 | 0 | -1 | -1 | -1 |
| **FirstAttendance_SpuriousEmergencyCare** | 1 | 0 | -1 | -1 | -1 |
| **Alri_Pneumonia_Treatment_Outpatient** | 3 | -1 | -1 | -1 | -1 |
| **Alri_Pneumonia_Treatment_Inpatient** | 3 | -1 | -1 | -1 | -1 |
| **Alri_Pneumonia_Treatment_Inpatient_Followup** | 3 | -1 | -1 | -1 | -1 |
| **BladderCancer_Investigation** | 3 | -1 | -1 | -1 | -1 |
| **BladderCancer_PalliativeCare** | 3 | -1 | -1 | -1 | -1 |
| **BladderCancer_Treatment** | 3 | -1 | -1 | -1 | -1 |
| **BreastCancer_Investigation** | 3 | -1 | -1 | -1 | -1 |
| **BreastCancer_PalliativeCare** | 3 | -1 | -1 | -1 | -1 |
| **BreastCancer_Treatment** | 3 | -1 | -1 | -1 | -1 |
| **CardioMetabolicDisorders_Investigation** | 3 | -1 | -1 | -1 | -1 |
| **CardioMetabolicDisorders_Prevention_CommunityTestingForHypertension** | 3 | -1 | -1 | -1 | -1 |
| **CardioMetabolicDisorders_Prevention_WeightLoss** | 3 | -1 | -1 | -1 | -1 |
| **CardioMetabolicDisorders_Treatment** | 3 | -1 | -1 | -1 | -1 |
| **AntenatalCare_FollowUp** | 3 | -1 | -1 | -1 | -1 |
| **AntenatalCare_Inpatient** | 3 | -1 | -1 | -1 | -1 |
| **AntenatalCare_Outpatient** | 3 | -1 | -1 | -1 | -1 |
| **AntenatalCare_PostAbortion** | 3 | -1 | -1 | -1 | -1 |
| **AntenatalCare_PostEctopicPregnancy** | 3 | -1 | -1 | -1 | -1 |
| **Contraception_Routine** | 3 | -1 | -1 | -1 | -1 |
| **Copd_Treatment** | 3 | -1 | -1 | -1 | -1 |
| **Depression_TalkingTherapy** | 3 | -1 | -1 | -1 | -1 |
| **Depression_Treatment** | 3 | -1 | -1 | -1 | -1 |
| **Diarrhoea_Treatment_Inpatient** | 3 | -1 | -1 | -1 | -1 |
| **Diarrhoea_Treatment_Outpatient** | 3 | -1 | -1 | -1 | -1 |
| **Epi_Adolescent_Hpv** | 2 | -1 | -1 | -1 | -1 |
| **Epi_Childhood_Bcg** | 2 | -1 | -1 | -1 | -1 |
| **Epi_Childhood_DtpHibHep** | 2 | -1 | -1 | -1 | -1 |
| **Epi_Childhood_MeaslesRubella** | 2 | -1 | -1 | -1 | -1 |
| **Epi_Childhood_Opv** | 2 | -1 | -1 | -1 | -1 |
| **Epi_Childhood_Pneumo** | 2 | -1 | -1 | -1 | -1 |
| **Epi_Childhood_Rota** | 2 | -1 | -1 | -1 | -1 |
| **Epi_Pregnancy_Td** | 2 | -1 | -1 | -1 | -1 |
| **Epilepsy_Treatment_Followup** | 3 | -1 | -1 | -1 | -1 |
| **Epilepsy_Treatment_Start** | 3 | -1 | -1 | -1 | -1 |
| **FirstAttendance_NonEmergency** | 3 | -1 | -1 | -1 | -1 |
| **Hiv_Prevention_Circumcision** | 2 | -1 | -1 | -1 | -1 |
| **Hiv_Prevention_Infant** | 2 | -1 | -1 | -1 | -1 |
| **Hiv_Prevention_Prep** | 2 | -1 | -1 | -1 | -1 |
| **Hiv_Test** | 2 | -1 | -1 | -1 | -1 |
| **Hiv_Treatment** | 2 | -1 | -1 | -1 | -1 |
| **DeliveryCare_Basic** | 3 | -1 | -1 | -1 | -1 |
| **DeliveryCare_Comprehensive** | 3 | -1 | -1 | -1 | -1 |
| **PostnatalCare_Maternal** | 3 | -1 | -1 | -1 | -1 |
| **PostnatalCare_Maternal_Inpatient** | 3 | -1 | -1 | -1 | -1 |
| **Malaria_Prevention_Iptp** | 2 | -1 | -1 | -1 | -1 |
| **Malaria_Test** | 2 | -1 | -1 | -1 | -1 |
| **Malaria_Treatment** | 2 | -1 | -1 | -1 | -1 |
| **Malaria_Treatment_Complicated** | 2 | -1 | -1 | -1 | -1 |
| **Measles_Treatment** | 3 | -1 | -1 | -1 | -1 |
| **PostnatalCare_Neonatal** | 3 | -1 | -1 | -1 | -1 |
| **PostnatalCare_Neonatal_Inpatient** | 3 | -1 | -1 | -1 | -1 |
| **OesophagealCancer_Investigation** | 3 | -1 | -1 | -1 | -1 |
| **OesophagealCancer_PalliativeCare** | 3 | -1 | -1 | -1 | -1 |
| **OesophagealCancer_Treatment** | 3 | -1 | -1 | -1 | -1 |
| **OtherAdultCancer_Investigation** | 3 | -1 | -1 | -1 | -1 |
| **OtherAdultCancer_PalliativeCare** | 3 | -1 | -1 | -1 | -1 |
| **OtherAdultCancer_Treatment** | 3 | -1 | -1 | -1 | -1 |
| **PostnatalCare_TreatmentForObstetricFistula** | 3 | -1 | -1 | -1 | -1 |
| **ProstateCancer_Investigation** | 3 | -1 | -1 | -1 | -1 |
| **ProstateCancer_PalliativeCare** | 3 | -1 | -1 | -1 | -1 |
| **ProstateCancer_Treatment** | 3 | -1 | -1 | -1 | -1 |
| **Rti_AcutePainManagement** | 3 | -1 | -1 | -1 | -1 |
| **Rti_BurnManagement** | 3 | -1 | -1 | -1 | -1 |
| **Rti_FractureCast** | 3 | -1 | -1 | -1 | -1 |
| **Rti_Imaging** | 3 | -1 | -1 | -1 | -1 |
| **Rti_MajorSurgeries** | 3 | -1 | -1 | -1 | -1 |
| **Rti_MedicalIntervention** | 3 | -1 | -1 | -1 | -1 |
| **Rti_MinorSurgeries** | 3 | -1 | -1 | -1 | -1 |
| **Rti_OpenFractureTreatment** | 3 | -1 | -1 | -1 | -1 |
| **Rti_ShockTreatment** | 3 | -1 | -1 | -1 | -1 |
| **Rti_Suture** | 3 | -1 | -1 | -1 | -1 |
| **Rti_TetanusVaccine** | 3 | -1 | -1 | -1 | -1 |
| **Schisto_MDA** | 3 | -1 | -1 | -1 | -1 |
| **Schisto_Treatment** | 3 | -1 | -1 | -1 | -1 |
| **Undernutrition_Feeding** | 3 | -1 | -1 | -1 | -1 |
| **Tb_Prevention_Ipt** | 2 | -1 | -1 | -1 | -1 |
| **Tb_Test_Clinical** | 2 | -1 | -1 | -1 | -1 |
| **Tb_Test_FollowUp** | 2 | -1 | -1 | -1 | -1 |
| **Tb_Test_Screening** | 2 | -1 | -1 | -1 | -1 |
| **Tb_Test_Xray** | 2 | -1 | -1 | -1 | -1 |
| **Tb_Treatment** | 2 | -1 | -1 | -1 | -1 |
| **Lowest priority considered** | 3 |  |  |  |  |

***“Reproductive, Maternal, Neonatal, and Child Health (RMNCH)” Policy***

Improving the country’s performance in reproductive, maternal, neonatal, and child health (RMNCH) has been a long-standing priority of the government of Malawi, as stated as early as in the HSSP I [8]. In the last two decades, the country has indeed made significant strides in reducing maternal, neonatal, infant, and particularly under five mortality rates [3]. Further efforts are however required to achieve target reductions set out in the Sustainable Development Goal 3 [9]. Under this prioritisation policy, perinatal appointments (including antenatal care, facility delivery, immediate and additional postnatal care for mothers and newborns) receive the highest-possible prioritisation, followed by appointments relevant to children’s health (fast- tracking children for: acute lower respiratory infections, diarrhoea, immunisation appointments, measles, schistosomiasis, and under-nutrition), and finally contraception appointments.

| **Treatment** | **Priority** | **If 5 years or under** | **If pregnant** | **If TB diagnosed** | **If HIV diagnosed** |
| --- | --- | --- | --- | --- | --- |
| **FirstAttendance_Emergency** | 1 | 0 | -1 | -1 | -1 |
| **FirstAttendance_SpuriousEmergencyCare** | 1 | 0 | -1 | -1 | -1 |
| **Alri_Pneumonia_Treatment_Outpatient** | 5 | 3 | -1 | -1 | -1 |
| **Alri_Pneumonia_Treatment_Inpatient** | 5 | 3 | -1 | -1 | -1 |
| **Alri_Pneumonia_Treatment_Inpatient_Followup** | 5 | 3 | -1 | -1 | -1 |
| **BladderCancer_Investigation** | 5 | -1 | -1 | -1 | -1 |
| **BladderCancer_PalliativeCare** | 5 | -1 | -1 | -1 | -1 |
| **BladderCancer_Treatment** | 5 | -1 | -1 | -1 | -1 |
| **BreastCancer_Investigation** | 5 | -1 | -1 | -1 | -1 |
| **BreastCancer_PalliativeCare** | 5 | -1 | -1 | -1 | -1 |
| **BreastCancer_Treatment** | 5 | -1 | -1 | -1 | -1 |
| **CardioMetabolicDisorders_Investigation** | 5 | -1 | -1 | -1 | -1 |
| **CardioMetabolicDisorders_Prevention_CommunityTestingForHypertension** | 5 | -1 | -1 | -1 | -1 |
| **CardioMetabolicDisorders_Prevention_WeightLoss** | 5 | -1 | -1 | -1 | -1 |
| **CardioMetabolicDisorders_Treatment** | 5 | -1 | -1 | -1 | -1 |
| **AntenatalCare_FollowUp** | 2 | -1 | -1 | -1 | -1 |
| **AntenatalCare_Inpatient** | 2 | -1 | -1 | -1 | -1 |
| **AntenatalCare_Outpatient** | 2 | -1 | -1 | -1 | -1 |
| **AntenatalCare_PostAbortion** | 2 | -1 | -1 | -1 | -1 |
| **AntenatalCare_PostEctopicPregnancy** | 2 | -1 | -1 | -1 | -1 |
| **Contraception_Routine** | 4 | -1 | -1 | -1 | -1 |
| **Copd_Treatment** | 5 | -1 | -1 | -1 | -1 |
| **Depression_TalkingTherapy** | 5 | -1 | -1 | -1 | -1 |
| **Depression_Treatment** | 5 | -1 | -1 | -1 | -1 |
| **Diarrhoea_Treatment_Inpatient** | 5 | 3 | -1 | -1 | -1 |
| **Diarrhoea_Treatment_Outpatient** | 5 | 3 | -1 | -1 | -1 |
| **Epi_Adolescent_Hpv** | 5 | -1 | -1 | -1 | -1 |
| **Epi_Childhood_Bcg** | 5 | 3 | -1 | -1 | -1 |
| **Epi_Childhood_DtpHibHep** | 5 | 3 | -1 | -1 | -1 |
| **Epi_Childhood_MeaslesRubella** | 5 | 3 | -1 | -1 | -1 |
| **Epi_Childhood_Opv** | 5 | 3 | -1 | -1 | -1 |
| **Epi_Childhood_Pneumo** | 5 | 3 | -1 | -1 | -1 |
| **Epi_Childhood_Rota** | 5 | 3 | -1 | -1 | -1 |
| **Epi_Pregnancy_Td** | 2 | -1 | -1 | -1 | -1 |
| **Epilepsy_Treatment_Followup** | 5 | -1 | -1 | -1 | -1 |
| **Epilepsy_Treatment_Start** | 5 | -1 | -1 | -1 | -1 |
| **FirstAttendance_NonEmergency** | 5 | -1 | -1 | -1 | -1 |
| **Hiv_Prevention_Circumcision** | 5 | -1 | -1 | -1 | -1 |
| **Hiv_Prevention_Infant** | 5 | -1 | -1 | -1 | -1 |
| **Hiv_Prevention_Prep** | 5 | -1 | -1 | -1 | -1 |
| **Hiv_Test** | 5 | -1 | -1 | -1 | -1 |
| **Hiv_Treatment** | 5 | -1 | -1 | -1 | -1 |
| **DeliveryCare_Basic** | 2 | -1 | -1 | -1 | -1 |
| **DeliveryCare_Comprehensive** | 2 | -1 | -1 | -1 | -1 |
| **PostnatalCare_Maternal** | 2 | -1 | -1 | -1 | -1 |
| **PostnatalCare_Maternal_Inpatient** | 2 | -1 | -1 | -1 | -1 |
| **Malaria_Prevention_Iptp** | 5 | -1 | -1 | -1 | -1 |
| **Malaria_Test** | 5 | -1 | -1 | -1 | -1 |
| **Malaria_Treatment** | 5 | -1 | -1 | -1 | -1 |
| **Malaria_Treatment_Complicated** | 5 | -1 | -1 | -1 | -1 |
| **Measles_Treatment** | 5 | 3 | -1 | -1 | -1 |
| **PostnatalCare_Neonatal** | 2 | -1 | -1 | -1 | -1 |
| **PostnatalCare_Neonatal_Inpatient** | 2 | -1 | -1 | -1 | -1 |
| **OesophagealCancer_Investigation** | 5 | -1 | -1 | -1 | -1 |
| **OesophagealCancer_PalliativeCare** | 5 | -1 | -1 | -1 | -1 |
| **OesophagealCancer_Treatment** | 5 | -1 | -1 | -1 | -1 |
| **OtherAdultCancer_Investigation** | 5 | -1 | -1 | -1 | -1 |
| **OtherAdultCancer_PalliativeCare** | 5 | -1 | -1 | -1 | -1 |
| **OtherAdultCancer_Treatment** | 5 | -1 | -1 | -1 | -1 |
| **PostnatalCare_TreatmentForObstetricFistula** | 2 | -1 | -1 | -1 | -1 |
| **ProstateCancer_Investigation** | 5 | -1 | -1 | -1 | -1 |
| **ProstateCancer_PalliativeCare** | 5 | -1 | -1 | -1 | -1 |
| **ProstateCancer_Treatment** | 5 | -1 | -1 | -1 | -1 |
| **Rti_AcutePainManagement** | 5 | -1 | -1 | -1 | -1 |
| **Rti_BurnManagement** | 5 | -1 | -1 | -1 | -1 |
| **Rti_FractureCast** | 5 | -1 | -1 | -1 | -1 |
| **Rti_Imaging** | 5 | -1 | -1 | -1 | -1 |
| **Rti_MajorSurgeries** | 5 | -1 | -1 | -1 | -1 |
| **Rti_MedicalIntervention** | 5 | -1 | -1 | -1 | -1 |
| **Rti_MinorSurgeries** | 5 | -1 | -1 | -1 | -1 |
| **Rti_OpenFractureTreatment** | 5 | -1 | -1 | -1 | -1 |
| **Rti_ShockTreatment** | 5 | -1 | -1 | -1 | -1 |
| **Rti_Suture** | 5 | -1 | -1 | -1 | -1 |
| **Rti_TetanusVaccine** | 5 | -1 | -1 | -1 | -1 |
| **Schisto_MDA** | 5 | 3 | -1 | -1 | -1 |
| **Schisto_Treatment** | 5 | 3 | -1 | -1 | -1 |
| **Undernutrition_Feeding** | 5 | 3 | -1 | -1 | -1 |
| **Tb_Prevention_Ipt** | 5 | -1 | -1 | -1 | -1 |
| **Tb_Test_Clinical** | 5 | -1 | -1 | -1 | -1 |
| **Tb_Test_FollowUp** | 5 | -1 | -1 | -1 | -1 |
| **Tb_Test_Screening** | 5 | -1 | -1 | -1 | -1 |
| **Tb_Test_Xray** | 5 | -1 | -1 | -1 | -1 |
| **Tb_Treatment** | 5 | -1 | -1 | -1 | -1 |
| **Lowest priority considered** | 5 |  |  |  |  |

***“Cardiometabolic disorders” (CMD) Policy***

The burden of non-communicable diseases (NCDs), including cardio-metabolic diseases (CMDs), is observed to be growing in low-income countries experiencing rapid societal transitions, including Malawi [10]. As these countries are already grappling with a significant burden of communicable diseases (CDs), the fear is that already strained health systems could become overwhelmed by a “double pandemic” of NCDs and CDs with a significant loss of life [11]. There is hope, however, that early intervention on NCDs in these settings could avert long-term health losses and costs for the health-sector. With this policy, therefore, we seek to assess the health impact of prioritising CMD-treatment at an early stage. The TLO simulation is uniquely equipped to assess this, as it comprehensively models both CDs and NCDs.

| **Treatment** | **Priority** | **If 5 years of under** | **If pregnant** | **If TB diagnosed** | **If HIV diagnosed** |
| --- | --- | --- | --- | --- | --- |
| **FirstAttendance_Emergency** | 1 | 0 | -1 | -1 | -1 |
| **FirstAttendance_SpuriousEmergencyCare** | 1 | 0 | -1 | -1 | -1 |
| **Alri_Pneumonia_Treatment_Outpatient** | 3 | -1 | -1 | -1 | -1 |
| **Alri_Pneumonia_Treatment_Inpatient** | 3 | -1 | -1 | -1 | -1 |
| **Alri_Pneumonia_Treatment_Inpatient_Followup** | 3 | -1 | -1 | -1 | -1 |
| **BladderCancer_Investigation** | 3 | -1 | -1 | -1 | -1 |
| **BladderCancer_PalliativeCare** | 3 | -1 | -1 | -1 | -1 |
| **BladderCancer_Treatment** | 3 | -1 | -1 | -1 | -1 |
| **BreastCancer_Investigation** | 3 | -1 | -1 | -1 | -1 |
| **BreastCancer_PalliativeCare** | 3 | -1 | -1 | -1 | -1 |
| **BreastCancer_Treatment** | 3 | -1 | -1 | -1 | -1 |
| **CardioMetabolicDisorders_Investigation** | 2 | -1 | -1 | -1 | -1 |
| **CardioMetabolicDisorders_Prevention_CommunityTestingForHypertension** | 2 | -1 | -1 | -1 | -1 |
| **CardioMetabolicDisorders_Prevention_WeightLoss** | 2 | -1 | -1 | -1 | -1 |
| **CardioMetabolicDisorders_Treatment** | 2 | -1 | -1 | -1 | -1 |
| **AntenatalCare_FollowUp** | 3 | -1 | -1 | -1 | -1 |
| **AntenatalCare_Inpatient** | 3 | -1 | -1 | -1 | -1 |
| **AntenatalCare_Outpatient** | 3 | -1 | -1 | -1 | -1 |
| **AntenatalCare_PostAbortion** | 3 | -1 | -1 | -1 | -1 |
| **AntenatalCare_PostEctopicPregnancy** | 3 | -1 | -1 | -1 | -1 |
| **Contraception_Routine** | 3 | -1 | -1 | -1 | -1 |
| **Copd_Treatment** | 2 | -1 | -1 | -1 | -1 |
| **Depression_TalkingTherapy** | 3 | -1 | -1 | -1 | -1 |
| **Depression_Treatment** | 3 | -1 | -1 | -1 | -1 |
| **Diarrhoea_Treatment_Inpatient** | 3 | -1 | -1 | -1 | -1 |
| **Diarrhoea_Treatment_Outpatient** | 3 | -1 | -1 | -1 | -1 |
| **Epi_Adolescent_Hpv** | 3 | -1 | -1 | -1 | -1 |
| **Epi_Childhood_Bcg** | 3 | -1 | -1 | -1 | -1 |
| **Epi_Childhood_DtpHibHep** | 3 | -1 | -1 | -1 | -1 |
| **Epi_Childhood_MeaslesRubella** | 3 | -1 | -1 | -1 | -1 |
| **Epi_Childhood_Opv** | 3 | -1 | -1 | -1 | -1 |
| **Epi_Childhood_Pneumo** | 3 | -1 | -1 | -1 | -1 |
| **Epi_Childhood_Rota** | 3 | -1 | -1 | -1 | -1 |
| **Epi_Pregnancy_Td** | 3 | -1 | -1 | -1 | -1 |
| **Epilepsy_Treatment_Followup** | 3 | -1 | -1 | -1 | -1 |
| **Epilepsy_Treatment_Start** | 3 | -1 | -1 | -1 | -1 |
| **FirstAttendance_NonEmergency** | 3 | -1 | -1 | -1 | -1 |
| **Hiv_Prevention_Circumcision** | 3 | -1 | -1 | -1 | -1 |
| **Hiv_Prevention_Infant** | 3 | -1 | -1 | -1 | -1 |
| **Hiv_Prevention_Prep** | 3 | -1 | -1 | -1 | -1 |
| **Hiv_Test** | 3 | -1 | -1 | -1 | -1 |
| **Hiv_Treatment** | 3 | -1 | -1 | -1 | -1 |
| **DeliveryCare_Basic** | 3 | -1 | -1 | -1 | -1 |
| **DeliveryCare_Comprehensive** | 3 | -1 | -1 | -1 | -1 |
| **PostnatalCare_Maternal** | 3 | -1 | -1 | -1 | -1 |
| **PostnatalCare_Maternal_Inpatient** | 3 | -1 | -1 | -1 | -1 |
| **Malaria_Prevention_Iptp** | 3 | -1 | -1 | -1 | -1 |
| **Malaria_Test** | 3 | -1 | -1 | -1 | -1 |
| **Malaria_Treatment** | 3 | -1 | -1 | -1 | -1 |
| **Malaria_Treatment_Complicated** | 3 | -1 | -1 | -1 | -1 |
| **Measles_Treatment** | 3 | -1 | -1 | -1 | -1 |
| **PostnatalCare_Neonatal** | 3 | -1 | -1 | -1 | -1 |
| **PostnatalCare_Neonatal_Inpatient** | 3 | -1 | -1 | -1 | -1 |
| **OesophagealCancer_Investigation** | 3 | -1 | -1 | -1 | -1 |
| **OesophagealCancer_PalliativeCare** | 3 | -1 | -1 | -1 | -1 |
| **OesophagealCancer_Treatment** | 3 | -1 | -1 | -1 | -1 |
| **OtherAdultCancer_Investigation** | 3 | -1 | -1 | -1 | -1 |
| **OtherAdultCancer_PalliativeCare** | 3 | -1 | -1 | -1 | -1 |
| **OtherAdultCancer_Treatment** | 3 | -1 | -1 | -1 | -1 |
| **PostnatalCare_TreatmentForObstetricFistula** | 3 | -1 | -1 | -1 | -1 |
| **ProstateCancer_Investigation** | 3 | -1 | -1 | -1 | -1 |
| **ProstateCancer_PalliativeCare** | 3 | -1 | -1 | -1 | -1 |
| **ProstateCancer_Treatment** | 3 | -1 | -1 | -1 | -1 |
| **Rti_AcutePainManagement** | 3 | -1 | -1 | -1 | -1 |
| **Rti_BurnManagement** | 3 | -1 | -1 | -1 | -1 |
| **Rti_FractureCast** | 3 | -1 | -1 | -1 | -1 |
| **Rti_Imaging** | 3 | -1 | -1 | -1 | -1 |
| **Rti_MajorSurgeries** | 3 | -1 | -1 | -1 | -1 |
| **Rti_MedicalIntervention** | 3 | -1 | -1 | -1 | -1 |
| **Rti_MinorSurgeries** | 3 | -1 | -1 | -1 | -1 |
| **Rti_OpenFractureTreatment** | 3 | -1 | -1 | -1 | -1 |
| **Rti_ShockTreatment** | 3 | -1 | -1 | -1 | -1 |
| **Rti_Suture** | 3 | -1 | -1 | -1 | -1 |
| **Rti_TetanusVaccine** | 3 | -1 | -1 | -1 | -1 |
| **Schisto_MDA** | 3 | -1 | -1 | -1 | -1 |
| **Schisto_Treatment** | 3 | -1 | -1 | -1 | -1 |
| **Undernutrition_Feeding** | 3 | -1 | -1 | -1 | -1 |
| **Tb_Prevention_Ipt** | 3 | -1 | -1 | -1 | -1 |
| **Tb_Test_Clinical** | 3 | -1 | -1 | -1 | -1 |
| **Tb_Test_FollowUp** | 3 | -1 | -1 | -1 | -1 |
| **Tb_Test_Screening** | 3 | -1 | -1 | -1 | -1 |
| **Tb_Test_Xray** | 3 | -1 | -1 | -1 | -1 |
| **Tb_Treatment** | 3 | -1 | -1 | -1 | -1 |
| **Lowest priority considered** | 3 |  |  |  |  |

***“Clinically Vulnerable” (CV) Policy***

Under this policy, all patients qualifying for one of the vulnerable categories considered (as described in section 2.3) will be eligible for fast-tracking to a “high priority” level for all types of services they might be seeking. All other individuals will instead be assigned a “low priority” level by default. We are, in particular, interested in establishing how health outcomes due to conditions related to vulnerable categories (i.e. causes of maternal and under-five mortality, TB, and AIDS) fare under this policy, compared to those that prioritise all relevant treatments regardless of patient’s characteristics.

| **Treatment** | **Priority** | **If 5 years or under** | **If pregnant** | **If TB diagnosed** | **If HIV diagnosed** |
| --- | --- | --- | --- | --- | --- |
| **FirstAttendance_Emergency** | 1 | 0 | -1 | -1 | -1 |
| **FirstAttendance_SpuriousEmergencyCare** | 1 | 0 | -1 | -1 | -1 |
| **Alri_Pneumonia_Treatment_Outpatient** | 3 | -1 | -1 | -1 | -1 |
| **Alri_Pneumonia_Treatment_Inpatient** | 3 | -1 | -1 | -1 | -1 |
| **Alri_Pneumonia_Treatment_Inpatient_Followup** | 3 | -1 | -1 | -1 | -1 |
| **BladderCancer_Investigation** | 3 | -1 | -1 | -1 | -1 |
| **BladderCancer_PalliativeCare** | 3 | -1 | -1 | -1 | -1 |
| **BladderCancer_Treatment** | 3 | -1 | -1 | -1 | -1 |
| **BreastCancer_Investigation** | 3 | -1 | -1 | -1 | -1 |
| **BreastCancer_PalliativeCare** | 3 | -1 | -1 | -1 | -1 |
| **BreastCancer_Treatment** | 3 | -1 | -1 | -1 | -1 |
| **CardioMetabolicDisorders_Investigation** | 2 | -1 | -1 | -1 | -1 |
| **CardioMetabolicDisorders_Prevention_CommunityTestingForHypertension** | 2 | -1 | -1 | -1 | -1 |
| **CardioMetabolicDisorders_Prevention_WeightLoss** | 2 | -1 | -1 | -1 | -1 |
| **CardioMetabolicDisorders_Treatment** | 2 | -1 | -1 | -1 | -1 |
| **AntenatalCare_FollowUp** | 3 | -1 | -1 | -1 | -1 |
| **AntenatalCare_Inpatient** | 3 | -1 | -1 | -1 | -1 |
| **AntenatalCare_Outpatient** | 3 | -1 | -1 | -1 | -1 |
| **AntenatalCare_PostAbortion** | 3 | -1 | -1 | -1 | -1 |
| **AntenatalCare_PostEctopicPregnancy** | 3 | -1 | -1 | -1 | -1 |
| **Contraception_Routine** | 3 | -1 | -1 | -1 | -1 |
| **Copd_Treatment** | 2 | -1 | -1 | -1 | -1 |
| **Depression_TalkingTherapy** | 3 | -1 | -1 | -1 | -1 |
| **Depression_Treatment** | 3 | -1 | -1 | -1 | -1 |
| **Diarrhoea_Treatment_Inpatient** | 3 | -1 | -1 | -1 | -1 |
| **Diarrhoea_Treatment_Outpatient** | 3 | -1 | -1 | -1 | -1 |
| **Epi_Adolescent_Hpv** | 3 | -1 | -1 | -1 | -1 |
| **Epi_Childhood_Bcg** | 3 | -1 | -1 | -1 | -1 |
| **Epi_Childhood_DtpHibHep** | 3 | -1 | -1 | -1 | -1 |
| **Epi_Childhood_MeaslesRubella** | 3 | -1 | -1 | -1 | -1 |
| **Epi_Childhood_Opv** | 3 | -1 | -1 | -1 | -1 |
| **Epi_Childhood_Pneumo** | 3 | -1 | -1 | -1 | -1 |
| **Epi_Childhood_Rota** | 3 | -1 | -1 | -1 | -1 |
| **Epi_Pregnancy_Td** | 3 | -1 | -1 | -1 | -1 |
| **Epilepsy_Treatment_Followup** | 3 | -1 | -1 | -1 | -1 |
| **Epilepsy_Treatment_Start** | 3 | -1 | -1 | -1 | -1 |
| **FirstAttendance_NonEmergency** | 3 | -1 | -1 | -1 | -1 |
| **Hiv_Prevention_Circumcision** | 3 | -1 | -1 | -1 | -1 |
| **Hiv_Prevention_Infant** | 3 | -1 | -1 | -1 | -1 |
| **Hiv_Prevention_Prep** | 3 | -1 | -1 | -1 | -1 |
| **Hiv_Test** | 3 | -1 | -1 | -1 | -1 |
| **Hiv_Treatment** | 3 | -1 | -1 | -1 | -1 |
| **DeliveryCare_Basic** | 3 | -1 | -1 | -1 | -1 |
| **DeliveryCare_Comprehensive** | 3 | -1 | -1 | -1 | -1 |
| **PostnatalCare_Maternal** | 3 | -1 | -1 | -1 | -1 |
| **PostnatalCare_Maternal_Inpatient** | 3 | -1 | -1 | -1 | -1 |
| **Malaria_Prevention_Iptp** | 3 | -1 | -1 | -1 | -1 |
| **Malaria_Test** | 3 | -1 | -1 | -1 | -1 |
| **Malaria_Treatment** | 3 | -1 | -1 | -1 | -1 |
| **Malaria_Treatment_Complicated** | 3 | -1 | -1 | -1 | -1 |
| **Measles_Treatment** | 3 | -1 | -1 | -1 | -1 |
| **PostnatalCare_Neonatal** | 3 | -1 | -1 | -1 | -1 |
| **PostnatalCare_Neonatal_Inpatient** | 3 | -1 | -1 | -1 | -1 |
| **OesophagealCancer_Investigation** | 3 | -1 | -1 | -1 | -1 |
| **OesophagealCancer_PalliativeCare** | 3 | -1 | -1 | -1 | -1 |
| **OesophagealCancer_Treatment** | 3 | -1 | -1 | -1 | -1 |
| **OtherAdultCancer_Investigation** | 3 | -1 | -1 | -1 | -1 |
| **OtherAdultCancer_PalliativeCare** | 3 | -1 | -1 | -1 | -1 |
| **OtherAdultCancer_Treatment** | 3 | -1 | -1 | -1 | -1 |
| **PostnatalCare_TreatmentForObstetricFistula** | 3 | -1 | -1 | -1 | -1 |
| **ProstateCancer_Investigation** | 3 | -1 | -1 | -1 | -1 |
| **ProstateCancer_PalliativeCare** | 3 | -1 | -1 | -1 | -1 |
| **ProstateCancer_Treatment** | 3 | -1 | -1 | -1 | -1 |
| **Rti_AcutePainManagement** | 3 | -1 | -1 | -1 | -1 |
| **Rti_BurnManagement** | 3 | -1 | -1 | -1 | -1 |
| **Rti_FractureCast** | 3 | -1 | -1 | -1 | -1 |
| **Rti_Imaging** | 3 | -1 | -1 | -1 | -1 |
| **Rti_MajorSurgeries** | 3 | -1 | -1 | -1 | -1 |
| **Rti_MedicalIntervention** | 3 | -1 | -1 | -1 | -1 |
| **Rti_MinorSurgeries** | 3 | -1 | -1 | -1 | -1 |
| **Rti_OpenFractureTreatment** | 3 | -1 | -1 | -1 | -1 |
| **Rti_ShockTreatment** | 3 | -1 | -1 | -1 | -1 |
| **Rti_Suture** | 3 | -1 | -1 | -1 | -1 |
| **Rti_TetanusVaccine** | 3 | -1 | -1 | -1 | -1 |
| **Schisto_MDA** | 3 | -1 | -1 | -1 | -1 |
| **Schisto_Treatment** | 3 | -1 | -1 | -1 | -1 |
| **Undernutrition_Feeding** | 3 | -1 | -1 | -1 | -1 |
| **Tb_Prevention_Ipt** | 3 | -1 | -1 | -1 | -1 |
| **Tb_Test_Clinical** | 3 | -1 | -1 | -1 | -1 |
| **Tb_Test_FollowUp** | 3 | -1 | -1 | -1 | -1 |
| **Tb_Test_Screening** | 3 | -1 | -1 | -1 | -1 |
| **Tb_Test_Xray** | 3 | -1 | -1 | -1 | -1 |
| **Tb_Treatment** | 3 | -1 | -1 | -1 | -1 |
| **Lowest priority considered** | 3 |  |  |  |  |

**References**

1. Connolly E, Mohan S, Twea P, Msuku T, Kees A, Sharma L, et al. Revision of Malawi’s Health Benefits Package: A Critical Analysis of Policy Formulation and Implementation. Value Health Reg Issues. 2023 Dec;39:84-94.

2. Mohan S, Walker S, Sengooba F, Kiracho EE, Mayora C, Ssennyonjo A, et al. Supporting the revision of the health benefits package in Uganda: A constrained optimisation approach. Health Economics. 2023 3;32(6):1244-55.

3. Government of the Republic of Malawi. Health Strategic Plan III: Reforming for Universal Health Coverage (First Edition); 2023. Available from:<https://www.health.gov.mw/download/hssp-iii/>.

4. Borghi J, Munthali S, Million LB, Martinez-Alvarez M. Health financing at district level in Malawi: an analysis of the distribution of funds at two points in time. Health Policy Plan. 2018 Jan;33(1):59-69.

5. Masefield SC, Msosa A, Grugel J. Challenges to effective governance in a low income healthcare system: a qualitative study of stakeholder perceptions in Malawi. BMC Health Serv Res. 2020;20(1142).

6. Sakala JJ, Chimatiro CS, Salima R, Kapachika A, Kalepa J, Stones W. The Integration of vertical and horizontal programmes for health systems strengthening in Malawi: a case study. Malawi Med J. 2022 Sep;34(3):206-12.

7. Nagemi C, Mwesigwa C. DONOR POWER AND PRIORITIZATION IN DEVELOPMENT ASSISTANCE FOR HEALTH POLICIES: THE CASE OF UGANDA. Journal of Developing Economies. 2021 Jan;2(1):54 74.

8. Government of the Republic of Malawi. Malawi Health Sector Strategic Plan 2011 - 2016: Moving towards equity and quality; 2011.

9. United Nations. Sustainable Development Goals. Accessed on the 12/09/2024 at<https://www.un.org/sustainabledevelopment/health/>.

10. Gowshall M, Taylor-Robinson SD. The increasing prevalence of non-communicable diseases in low-middle income countries: the view from Malawi. Int J Gen Med. 2018;11:255-64.

11. World Health Organization. Global Action Plan for the Prevention and Control of Noncommunicable Diseases 2013–2020; 2013. Available from:<https://www.who.int/publications/i/item/9789241506236>.
