# Supplementary material for: A new approach to Health Benefits Package design: an application of the Thanzi La Onse model in Malawi
Source: PLoS Comput Biol. 2024 Sep 30;20(9):e1012462. doi: 10.1371/journal.pcbi.1012462 (PMC11567512; doi:10.1371/journal.pcbi.1012462)
Supplement: S4 Appendix — (DOCX) [file pcbi.1012462.s004.docx]

**Understanding the relative performance of di**ff**erent prioritisation policies in key areas of health**

In this section we review in detail the performance of different policies under some of the leading causes of DALYs over the simulated period, namely lower respiratory infections, AIDS, neonatal disorders, malaria, Tb, and measles.

**Lower respiratory infections.** In Fig A we show the DALYs incurred per year due to this cause (top plot), together with the number of related HSIs delivered under different policies, broken down by appointment type and level at which they were delivered (bottom plot).

The best-performing policy under lower respiratory infections is LCOA, which delivers the highest volume of related HSI delivered. Under this policy, outpatient pneumonia appointments are allocated high priority, but inpatient appointments are assigned low priority; it therefore may be quite surprising that a higher rate of inpatient appointments is being delivered under this policy at level 2 compared to policies that deliberately prioritise these appointments, such as the RMNCH and the CV policy. This is due to the fact that LCOA, unlike any of the other policies considered (except for the HSSP-III HBP) deliberately excludes from its provision several of the treatments taking place at this level, such as appointments related to cardiometabolic disorders — which constitute some of the most in demand treatment at this level — all of cancer care, and major surgeries linked to road traffic incidents, among others. This frees up a large amount of resources destined for low-priority treatments at level 2, which can therefore be allocated to key but low priority (according to the LCOA) treatments such as pneumonia inpatient appointments, allowing it to make significant improvements in one of the most important areas of health. As most high-priority appointments under LCOA are delivered at level 1a (although, thanks to the exclusion of HIV treatment, the competition for resources among high-priority appointments at level 1a is not as intense as under other policies) at this level highly prioritised services ALRI (such as pneumonia outpatient appointments) are delivered at a higher rate than for RMNCH and CV, but de-prioritised ones (such as inpatient follow-up) struggle to be delivered. This perfectly illustrates how the stricter selection of high-priority appointments under LCOA as well as its more nuanced prioritisation strategy results in a higher volume of service delivery in key areas of health.

As a leading cause of DALYs incurred among children and infants, many of the request for pneumonia-related treatments will be made by children under five-year, which are highly prioritised by the CV policy and RMNCH. The former indeed is the second highest performing in this area of health. Unlike LCOA, CV is not able to deliver as many appointments despite placing high priority on them, likely because it is overwhelmed by demand for other highly- prioritised services, such as e.g. delivering 4-5 million of HIV-related HSIs a year at level 1a, or perinatal appointments at level 2. RMNCH, on the other hand, while reserving the second-tier prioritisation for these treatments, isn’t able to deliver as high a volume of treatments due to the overwhelming demand for perinatal treatments both at level 1a and 2, as will be discussed next. VP and CMD both allocate resources elsewhere, hence they have the lowest rate of service delivery. They however don’t perform significantly worse than the NP and HSSP-III HBP, which have the lowest rate of service delivery. The incidence of risk factors across different policies are the likely explanation for this: thanks to its effective delivery of childhood pneumonia vaccinations (see Fig F, bottom plot) and performance in HIV (given that HIV is the most severe risk factor for lower respiratory infections), VP performs as well as NP despite delivering half the number of appointments. CV on the other hand has a worse AIDS performance, but delivers a much higher number of measles treatments than both VP and HSSP-III HBP, since the latter excludes measles treatment completely. Differences in vaccination rates could explain the remaining differences.


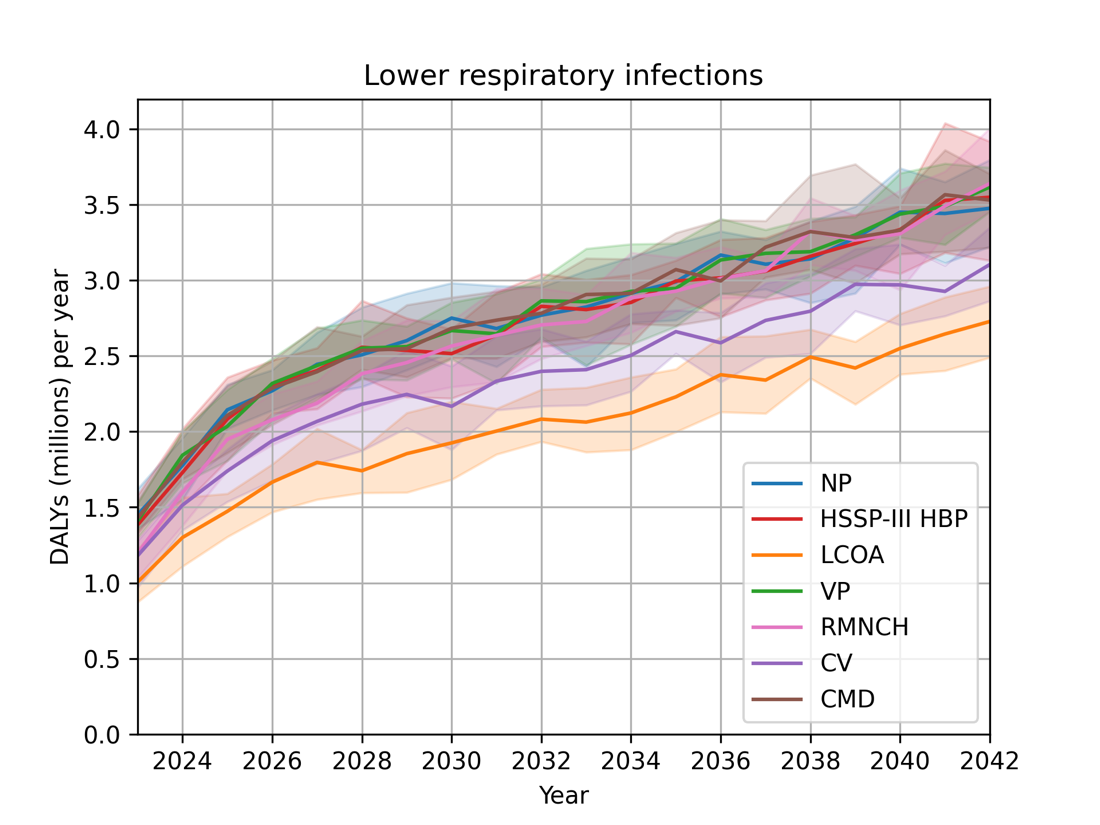


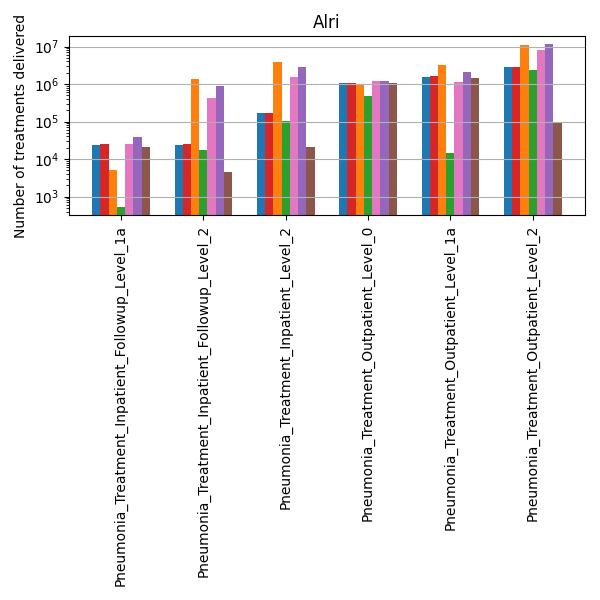


**Fig A.** Yearly breakdown of ALRI-caused DALYs (top plot), and ALRI-specific services delivered over the entire period.

**AIDS.** In Fig B (top row) we compare the evolution of AIDS-caused DALYs under each policy (left plot), as well as the demand for and delivery of HIV-related care in each case (right plot). We see that the transition to a resource-constrained healthcare system results in a spike in AIDS-caused DALYs: as the “rigid healthcare-system” struggles to meet the high demand for HIV services, patients will be unable to access treatment, leading to both a raised risk of AIDS and a higher incidence of HIV in the population. While the initial spike in AIDS-caused DALYs is common across all policies, it is far more prominent for RMNCH and LCOA, where it results in an excess of over four million DALYs the first three years. For the RMNCH policy, this is driven by the fact that all HIV-related HSIs — including prevention, testing, and treatment — are de-prioritised in favour of other services, leading to the lowest overall rate of HIV-related service delivery observed, roughly less than half that of other policies. In the case of LCOA, the increase in AIDS-related DALYs is instead driven — despite the very high rate of delivery of HIV-prevention and testing services, which is comparable to other policies — by the complete exclusion of HIV treatment from the health-care provision under this policy, as shown in the middle row of Fig B.

The initial increase in mortality in the first three years (2023–2025) leads to a consequent drop in the incidence and prevalence of the disease in the following years. This results in RMNCH and LCOA policies actually incurring slightly fewer DALYs per year due to AIDS compared to the NP between 2027 and 2032. Whereas other policies at this point continue to make progress in DALYs due to this cause, the lack of treatment by these two causes results in stagnating rates of AIDS-caused DALYs. This is in part driven by the low rate of treatment among women, which leads to a surge in AIDS-related DALYs among infants in later years (see bottom row of Fig B). Notice that, although HIV infant prevention is prioritised by LCOA and its rate of delivery is higher than for any other policy, this is only 30 % effective compared to a 100 % prevention in mother-to-child transmission during pregnancy, labour and delivery, and throughout breastfeeding when pregnant women are under treatment.

Among policies which sought to deliberately prioritise HIV treatment (i.e. CV and VP) the marginally higher rates of treatment delivery succeed in preventing ∼ 0.4 million DALYs a year compared to the NP case at the peak of the infection surge in 2025 and 2026, resulting in a lower cumulative number of DALYs as a result of AIDS over the entire period. HSSP-III HBP deliberately excludes circumcision and PrEP from its provision, however due to the low uptake

of these services this doesn’t seem to significantly affect the incidence of the disease, such that the performance of this policy overall closely traces that of NP, except for a small reduction in the average peak DALYs in 2026, possibly due to the very small excess in treatments delivered thanks to the exclusion of circumcision and PrEP services competing at the same level.


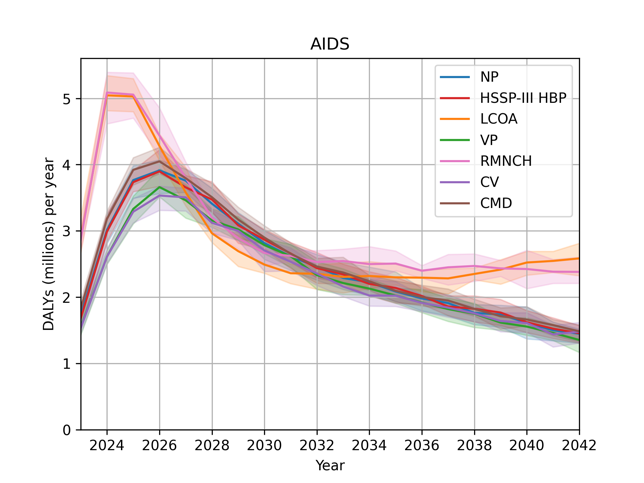

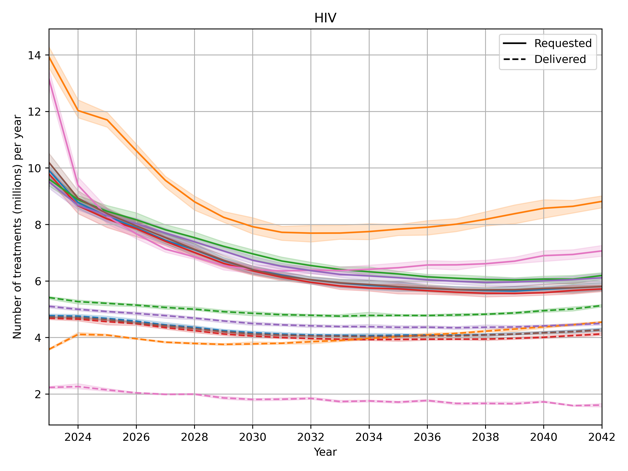


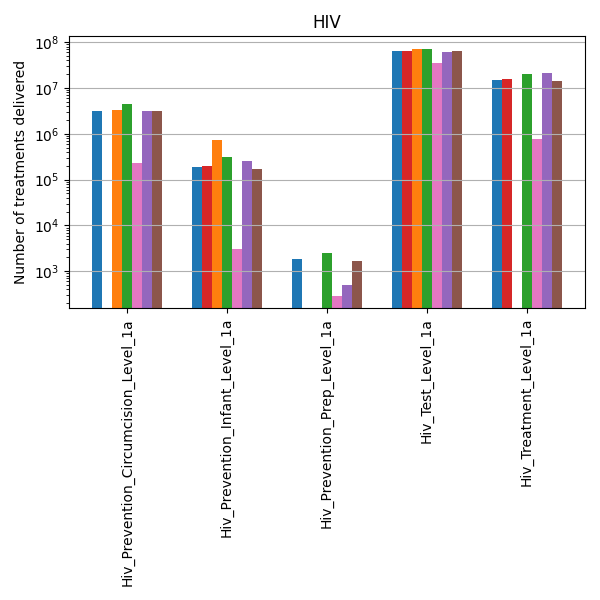


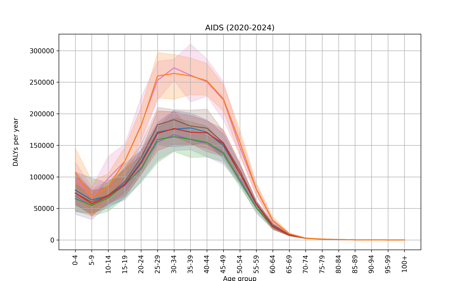

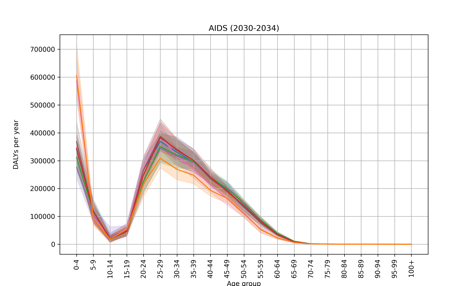

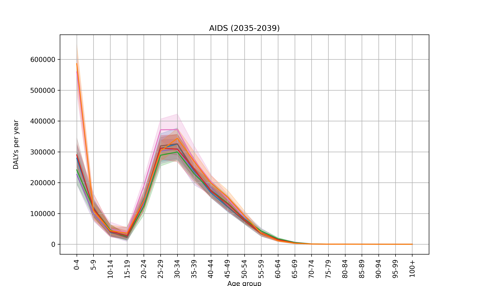


**Fig B.** *Top row*: Yearly breakdown of AIDS-caused DALYs (left plot), and HIV-specific services requested and delivered under different prioritisation policies over the simulated policy-implementation period. Middle row: Breakdown of mean delivered HIV-related HSIs for different prioritisation policies in the same period (2023-2042 inclusive). Bottom row: Evolution of AIDS-caused DALYs incurred in different age groups in different five-year intervals during the policies implementation. Despite prioritising HIV Prevention for infants, we notice an increase in instances among infants in the LCOA and RMNCH policies due to the low rate of treatment in the population, and hence among pregnant women.

**Neonatal disorders.** In Fig C we show the yearly DALYs incurred due to neonatal disorders under different policies, and the number of antenatal, delivery, and postnatal appointments delivered in each case.

Neonatal disorders constitute the third leading cause of DALYs overall, and represent one of the areas of health most focused on by the RMNCH policy, which prioritises perinatal care above all other treatments. The most noticeable outcome is that, despite the huge effort made by the


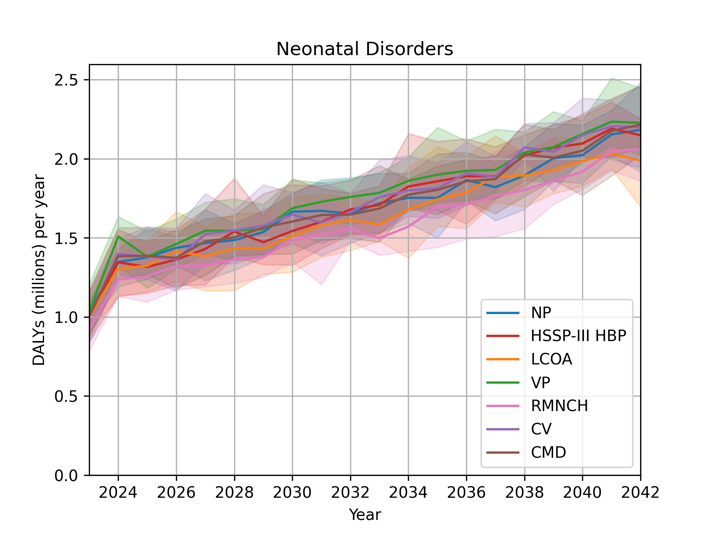

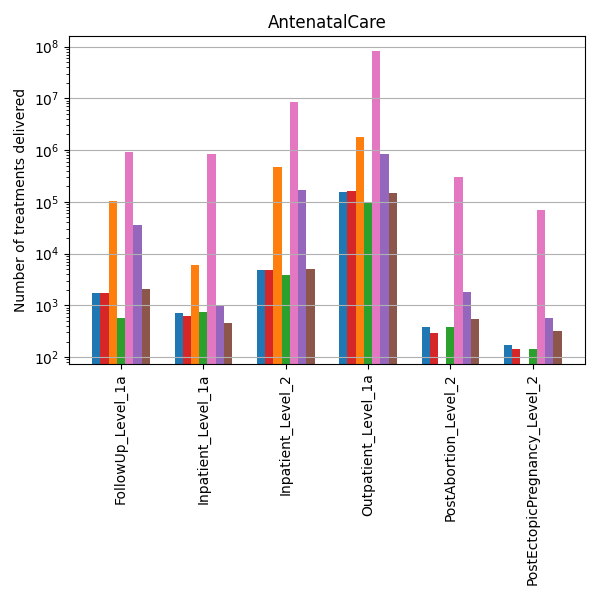

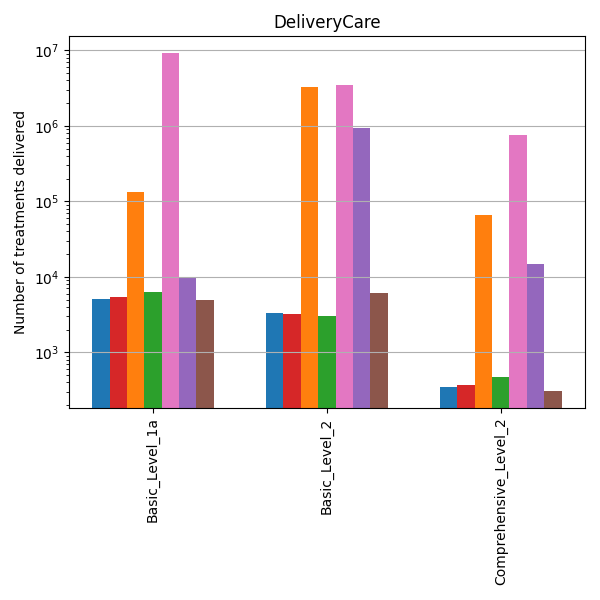

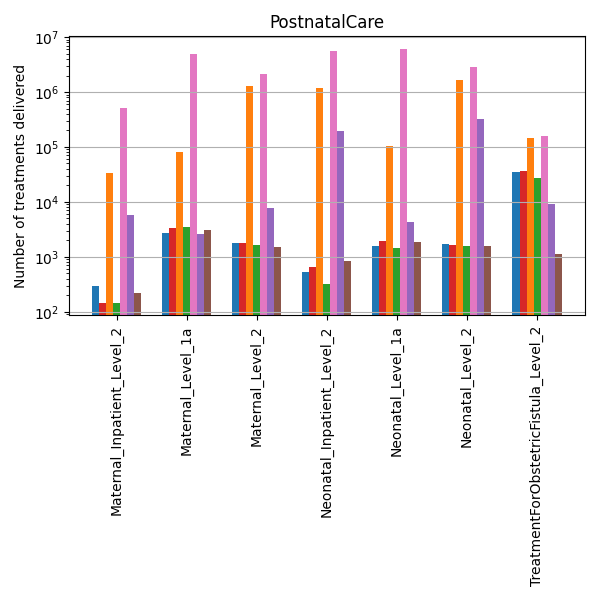


**Fig C.** *Top left plot:* Yearly DALYs incurred by different policies due to neonatal disorders. In the remaining plots we show the total number of HSIs delivered in the same period for antenatal care (top right plot), delivery care (bottom left plot), and postnatal care (bottom right plot). The difference in the delivery rates of these HSIs under different policies has little impact on the health burden incurred due to these conditions.

RMNCH policy in delivering a large volume of perinatal services, the improvement in neonatal outcomes is marginal at best. A number of important factors hinder the translation of perinatal care delivered into health gains for newborns and mothers, as discussed in detail in [1]: access to antenatal services is crucial to prevent important risk factors (such as anaemia and maternal malaria infection) which increase the probability of incurring premature labour, one of the main contributors to neonatal deaths. Rates of health-seeking for antenatal appointments are however predominantly low, which means that even under a significant scale up of the programme under RMNCH only a fraction of women who could have benefited from these services are reached: only 51% of women receive four or more ANC visit, while only 24% of women attend their first ANC visit within the first trimester as visits are clustered later in pregnancy, limiting the effectiveness of treatment overall [2]. Furthermore, the need for vital services such as resuscitation of newborns is assumed to be identified in only 60% of cases based on observed clinical quality; even if delivered, treatment reduces the probability of neonatal death due to commonly occurring respiratory conditions by only 37%; in addition, we assume treatment is less effective for preterm neonates experiencing preterm respiratory distress syndrome, in which treatment only reduces the probability of death by 19% [1]. These effects combined therefore result in a sub-optimal health return from perinatal services being prioritised.

Despite the poor impact that these services have on a population-wide scale, interesting considerations can be made about the different rates of service delivery across different policies. As RMNCH prioritises perinatal appointments above all others, all appointment types have a high volume of delivered treatments. LCOA and CV however both assign high priority to these appointments — although in the case of LCOA, Post-Ectopic pregnancy appointments and post- abortion care are completely excluded from the healthcare service provision, while inpatient antenatal care is assigned low priority. For LCOA, the lower number of high priority services at level 2 and the exclusion of HIV treatment at level 1a means that resources are more readily available at these levels to deliver these services, resulting in a much higher rate of service delivery for LCOA than for the CV policy.

**Malaria.** In Fig D we show the yearly DALYs incurred due this cause (top plot) as well as the number of related treatments delivered (middle plot) and requested (bottom plot) under each policy. From a DALY perspective, the LCOA shows the best performance overall, with VP closely following. While both policies place high priority on malaria-related services, the ability of VP to deliver malaria-related treatments is limited by its high prioritisation of HIV treatments at level 1a, which are instead completely excluded from its provision by the LCOA. At level 2, the only competing high-priority HSIs for VP are Epi appointments, and the relative rate of appointment delivery between the two policies merely reflects the relative request for treatment (see bottom plot). As under LCOA a higher rate of Intermittent Preventive Treatment of Pregnant women (IPTP) is delivered, the lower request for complicated treatment at level 2 may simply be a result of better coverage of the most vulnerable individuals at risk of malaria complications, i.e. small children. CV, which prioritises children in all health care, is similarly able to deliver high rates of malaria treatment and therefore prevent a lot of burden, however it is further limited compared to the VP policy by its further prioritisation, in addition to HIV and Tb, of HSIs involving perinatal care and children’s health. Note that RMNCH does not prioritise malaria treatment for children, and the overwhelming prioritisation of perinatal appointments means that very few resources are available to attend to malaria services.


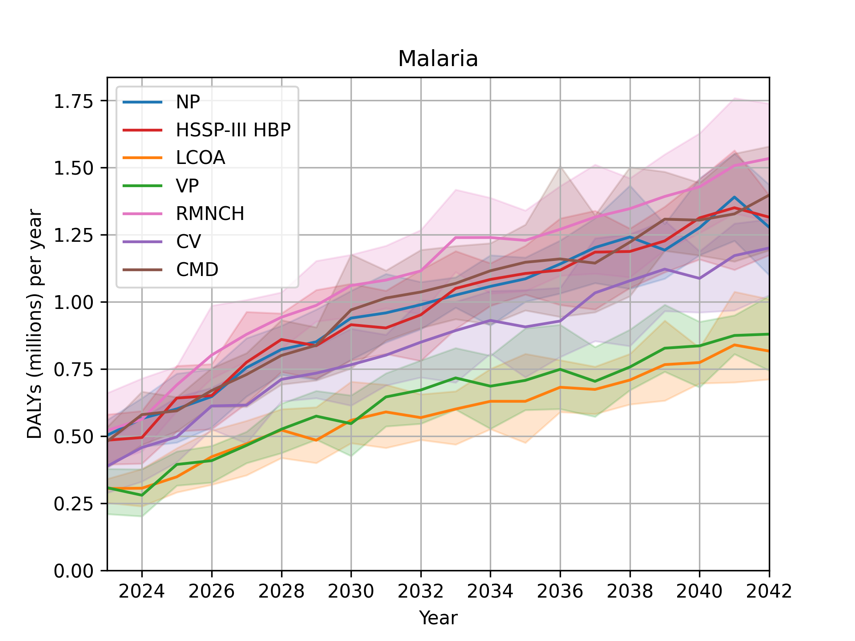


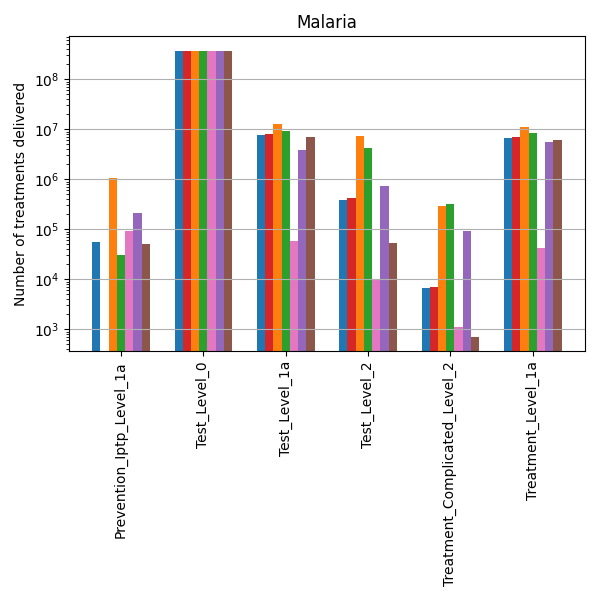


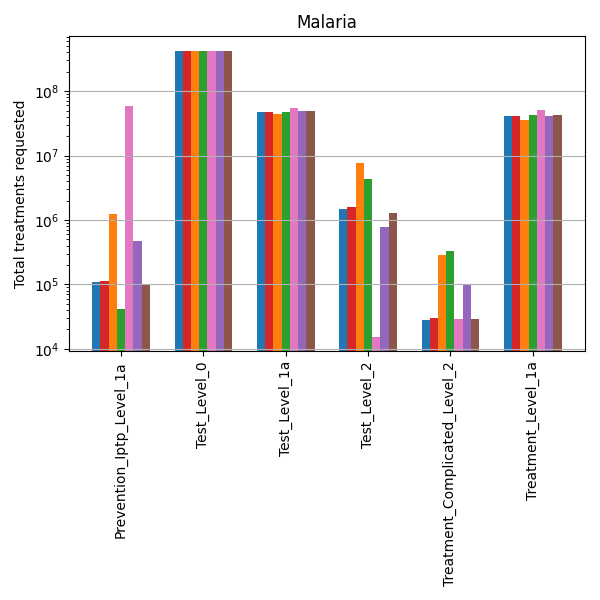


**Fig D**. Yearly DALYs caused by malaria *(top-plot)*, total delivered disease-specific HSIs *(middle plot)*, and total requested disease-specific HSIs *(bottom plot)*.

**Tb.** In Fig E we show the yearly DALYs incurred due this cause (top plot) as well as the number of related treatments delivered (middle plot) and requested (bottom plot) under each policy. Once again, LCOA is the best-performing policy in this area, thanks to its highest rate of service delivery. The request for TB screening (see Fig E) — which starts the TB referral pathway — is comparable across all policies, however the LCOA policy — which has more resources available at level 1a due to its more targeted prioritisation strategy — is the most able to deliver on this demand for screening, providing an additional 1–2 million extra TB screening services a year compared to e.g. Vertical Programmes, as well as satisfy all subsequent appointments (notice that the only treatment which has a lower rate of delivery, Follow-up appointments, are assigned a lower priority under the LCOA strategy). The lower request for preventative Ipt treatments under the LCOA and RMNCH is likely the result of a lower cumulative incidence of HIV due to higher initial mortality rates.

**Measles.** In Fig F we show the yearly DALYs incurred due this cause (top plot) as well as the number of related treatments delivered (middle plot). We observe that, for this cause of DALY, the performance of each policy seems to poorly correlated to the number of treatments delivered. For example, while VP and CV have a wildly different rate of treatment delivery, the incurred DALYs are broadly similar, while LCOA, which delivers an amount of treatment intermediate between the two, performs far better. This is due to the TLO simulation accurately capturing both the impact of risk factors — namely lack of measles vaccination — as well as the low efficacy of measles treatment, which results in the DALYs incurred due to measles being primarily driven by the relevant vaccination rates, rather than treatment itself, as the former protects against the most serious manifestation of the disease. There are indeed no significant differences in incurred DALYs due to measles between the NP policy and the HSSP-III HBP — which deliver a similar rate of vaccination — despite the latter completely excluding measles treatment from its provision and the former delivering two million measles treatment appointments overall. For the remaining policies (RMNCH, LCOA, VP, and CV) their relative performance exactly correlates with its vaccination rate.


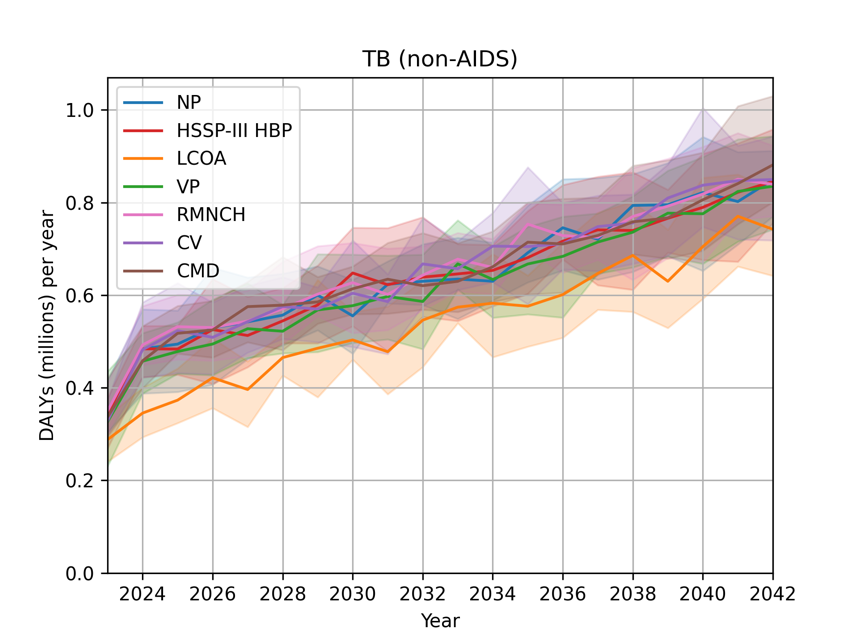

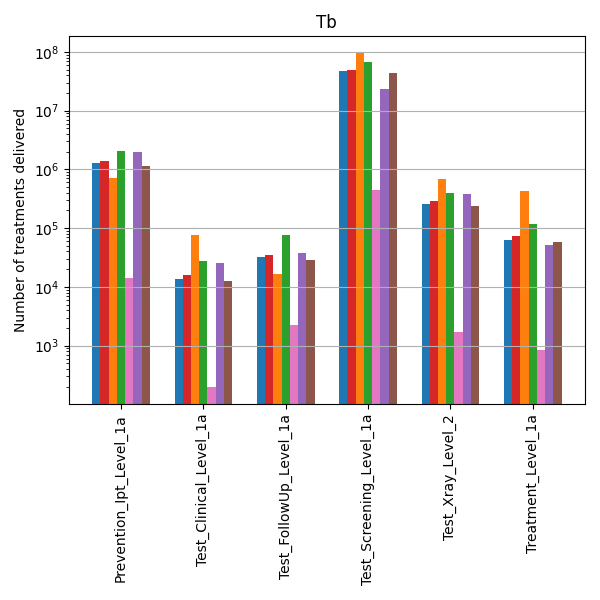


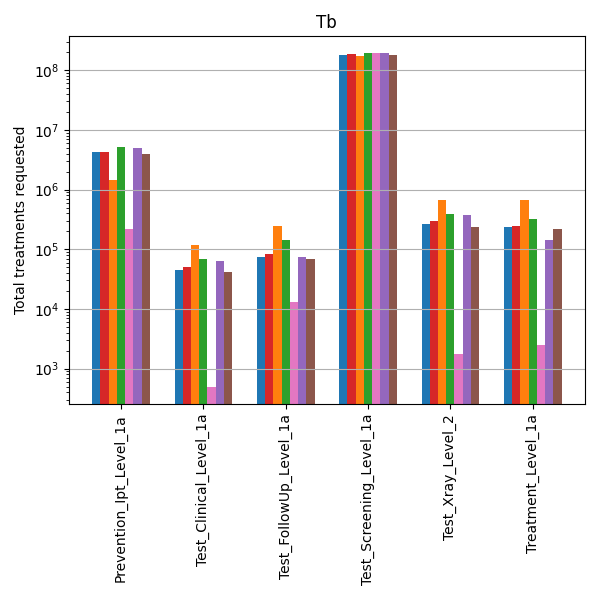


**Fig E**. DALYs incurred per year due to TB (top plot), as well as the total related appointments delivered (middle plot) and requested (bottom plot).


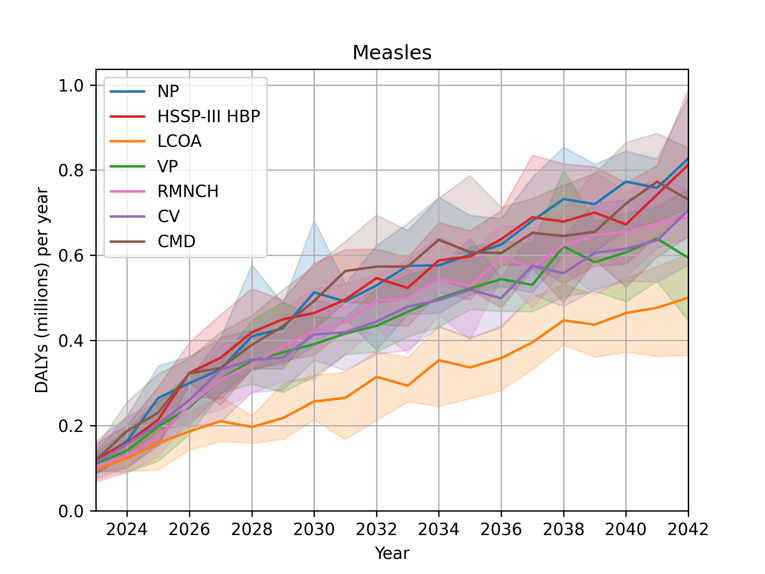


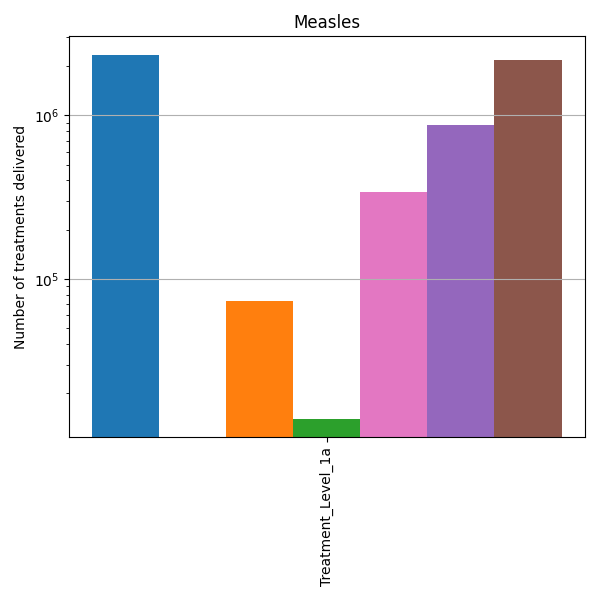


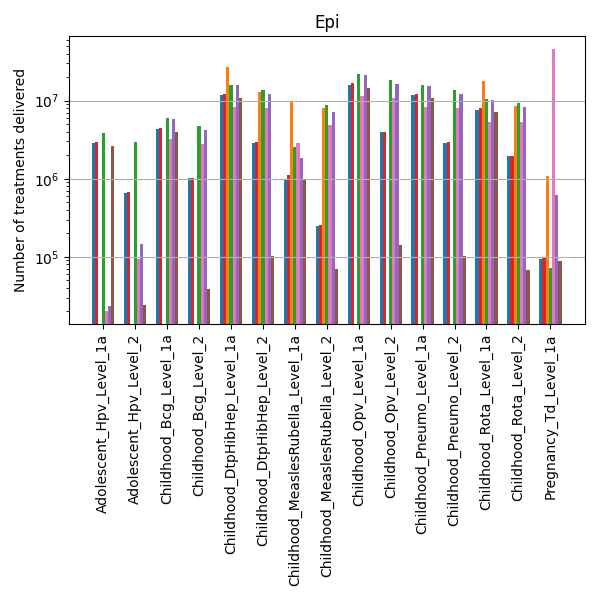


**Fig F**. Number of yearly DALYs incurred due to measles (top plot), number of measles treatments delivered (middle plot), and number of vaccinations delivered (bottom plot) under different policies.

**References**

1. Collins J, Allott H, Ng’ambi W, Li Lin I, Giordano M, Graham M, et al.. Estimating the impact of maternity service delivery on health in Malawi: An individual-based modelling study; 2024. Preprint. Available from:<https://doi.org/10.21203/rs.3.rs-3937439/v1>.

2. National Statistical Office, Malawi and ICF. Malawi Demographic and Health Survey 2015-16; 2017. Available from: [https://dhsprogram.com/pubs/ pdf/FR319/FR319.pdf](https://dhsprogram.com/pubs/pdf/FR319/FR319.pdf).
