# Supplementary material for: A new approach to Health Benefits Package design: an application of the Thanzi La Onse model in Malawi
Source: PLoS Comput Biol. 2024 Sep 30;20(9):e1012462. doi: 10.1371/journal.pcbi.1012462 (PMC11567512; doi:10.1371/journal.pcbi.1012462)
Supplement: S5 Appendix — (DOCX) [file pcbi.1012462.s005.docx]

**Accounting for diverging population sizes across the evaluated policies**

One of the difficulties in assessing the relative performance of different policies arises from the fact that, as birth rates and mortality rates in different scenarios diverge, the populations in different scenarios may start to look increasingly different. In Fig A (top plot), we show the population size over the simulated period for different policies. We see that LCOA, the best performing policy over the simulated 20-year period, is also the one with the smallest population by the end of this period, with a 5% smaller population than the policy with the largest population size in 2042, VP. A main driver of this is the fact that LCOA is one of the only policies that effectively prioritises contraception appointments. This is reflected in the population structure at the end of 2042, which highlights how differences in population sizes are primarily due to differences in number of infants and children. If we want to assess the relative performance of the healthcare system as it caters with the same finite resources to populations of different sizes, we may therefore reconsider whether a health system that incurs fewer DALYs but also serves a smaller population can really be said to be performing better than one that incurs more DALYs while serving a larger population size with the same resources.

To address this, in Fig B, we show yearly DALYs normalised by population size incurred under different policies averaged over the entire simulated period (2023–2042). We would expect policies which prevented births to perform relatively worse than under the unnormalised DALY metric; indeed, we see that, especially in the initial five-year period, the relative performance of LCOA compared to a NP is marginally decreased (see Fig 6 for comparison) however the relative performance of the policies overall is mostly unaffected. The error bars show a 95% CI, while the blue shaded area extends the NP’s interval to facilitate comparison.


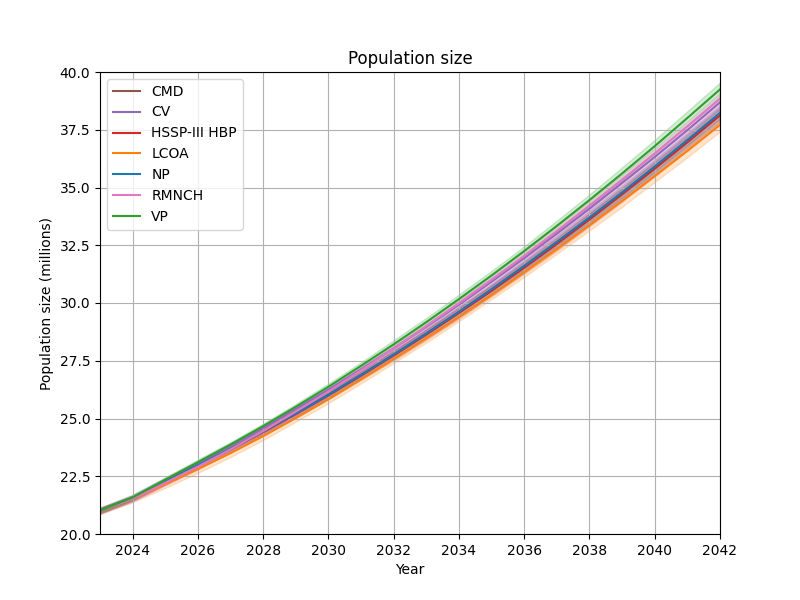

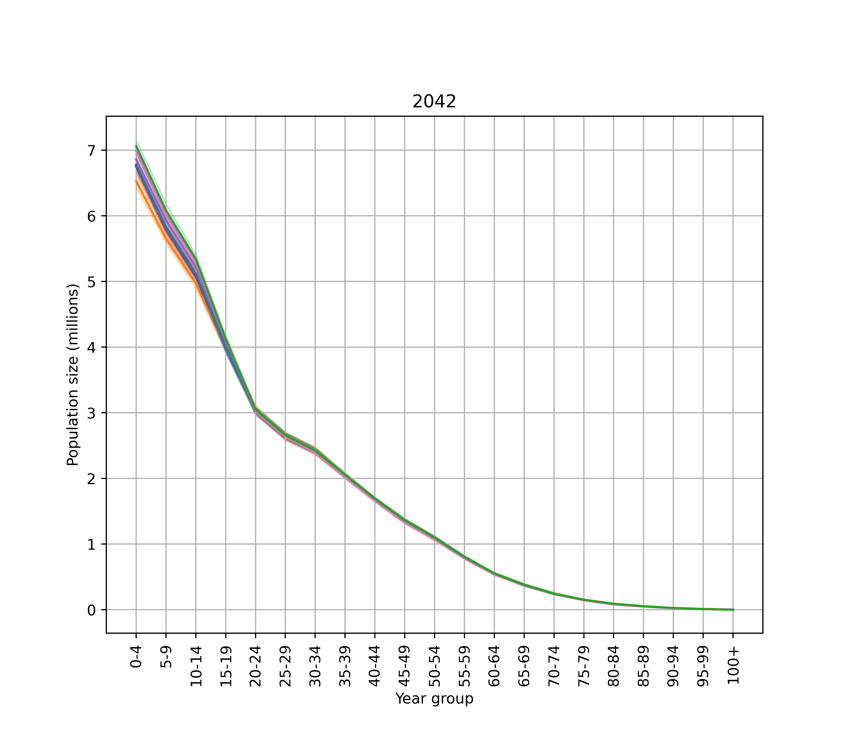


**Fig A**. *Top plot:* Population size over the simulated period for different policies. *Bottom plot:* Breakdown of population by age group at the end of the simulated period.


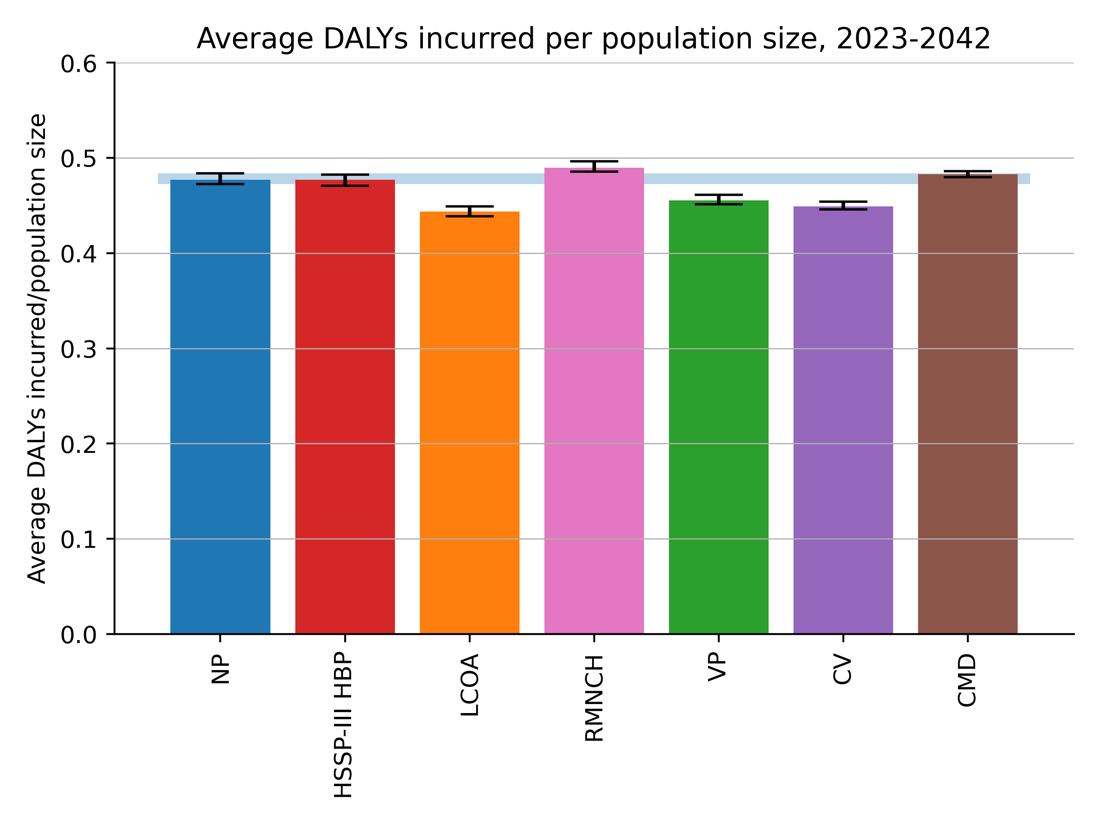

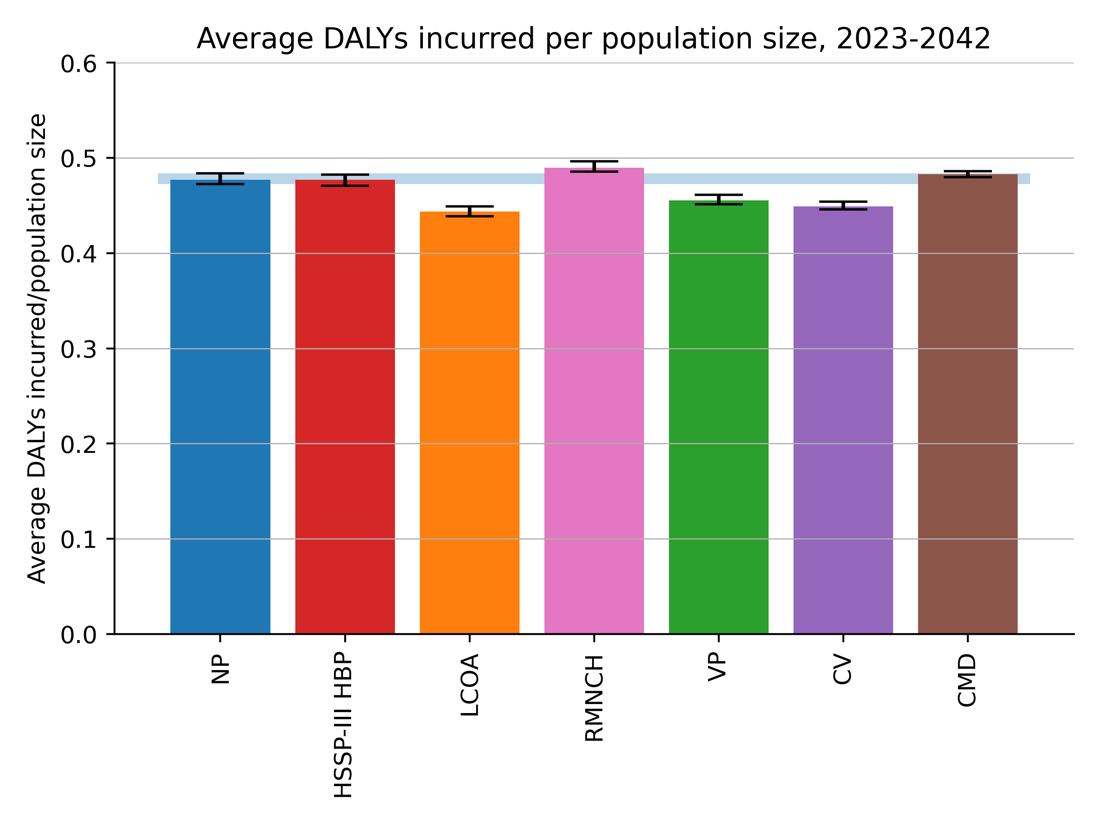


**Fig B**. Average yearly DALYs normalised by population size each year for different policies, during the first five years of policy implementation (top plot) or for the entire 20-year period (bottom plot).
