# Supplementary material for: A new approach to Health Benefits Package design: an application of the Thanzi La Onse model in Malawi
Source: PLoS Comput Biol. 2024 Sep 30;20(9):e1012462. doi: 10.1371/journal.pcbi.1012462 (PMC11567512; doi:10.1371/journal.pcbi.1012462)
Supplement: Attachment — Submitted filename: Response_to_reviewers.pdf [file pcbi.1012462.s010.pdf]

We would like to thank the referees for their time and effort in reviewing our responses to their comments and revisions to the draft. We would also like to thank them again for allowing us to greatly improve the quality of this manuscript through this review process.

We provide a detailed reply to each comment below:

Reviewer #3:

Comment 1. I have noted a few grammatical suggestions.
Response: These have been implemented on lines: 20, 38, 155, 169, 201, 216, 240, 250. At present we have kept the spelling as British English, but would be happy to revise the entire draft into American English should the Editor require it.

Comment 2. I think you should add a paragraph or two about whether this approach could be used when reinfections occur -- e.g., schistosomiasis
Response: We have added the following paragraph to the manuscript on line 116: ”The simulation accounts for the possibility of reinfection among individuals who have recovered from the disease before, and considers the impact of prior treatment and/or natural immunity on both the likelihood of reinfection and potential outcomes.”

In addition, the new version of the manuscript now includes:

- A formal acknowledgment of the contribution of the referees to the manuscript in the Acknowledgement section.
- A revised version of the data availability statement, which now includes a link to a Zenodo repository from which post-processed output data can be directly accessed, in addition to the GitHub link from which the data can be reproduced.
- A clarification that some of the Figures cited are contained in the supplementary materials, and a correction as to which supplementary material these are contained in (lines 397 and 520).
