# Supplementary material for: A new approach to Health Benefits Package design: an application of the Thanzi La Onse model in Malawi
Source: PLoS Comput Biol. 2024 Sep 30;20(9):e1012462. doi: 10.1371/journal.pcbi.1012462 (PMC11567512; doi:10.1371/journal.pcbi.1012462)
Supplement: Attachment — Submitted filename: MS PCOMPBIOL00243 review.docx [file pcbi.1012462.s006.docx]

MS Review PCOMPBIOL-00243

A new approach to Health Benefits Package design: an application of the Thanzi La Onse model in Malawi

The paper nicely lays out the shortcomings of cost-effectiveness data, rightly calls for modesty in generalising such data across settings and timeframes, and presents an interesting novel approach to health service prioritisation.

The structure of the paper is a bit atypical: usually the study objectives are placed at the end of the introduction (before the methods section), but in this paper the study aim comes at the end of the methods section (lines 230-234). I personally have no objection, and leave it to the Editor(s) to decide whether this is acceptable. I would however recommend a specification of the dual study aim (2x “We seek to …”) to better align them with the 4 subsections in Results.

Also in relation to structure, section 3.3 (What makes a prioritisation strategy successful? – with 4 success factors) appears to be more something like a discussion. Please consider moving this from the Results section to Discussion.

I suggest to shorten the Conclusions section. Repetition of things that have been stated earlier in the paper (which takes up the first half of this section) needs to be avoided in any case.

My comments are as follows.

1. Some strong statements are made in the Abstract, without sufficient supporting references in the paper. For instance, (a) “Currently available methods to guide policymaking are limited and rely upon strong assumptions.” – it does not become clear (from the Introduction or Discussion) which methods are being referred to and which sources in particular justify this claim?; and (b) “This strategy instead offers consistent health gains on a year-to-year basis and a 5% reduction in DALYs overall, which suggests a more patient-centred approach in future might prove beneficial.” – ‘instead’ of what? ‘More patient-centred’ than which other approach? The term ‘patient-centred’ may mislead the reader. I believe it’s important to add approach ‘to prioritisation’; or say ‘service prioritisation based on patient characteristics, rather than service types’.
2. The paper does refer to commonly used methods and databases on the cost-effectiveness of health services/ interventions (such as DCP, TUFTS CE registry, WHO-CHOICE; the UHC Compendium would also deserve mentioning) but it doesn’t sufficiently acknowledge recent guidance documents and literature on country experiences with extensive stakeholder deliberation to inform HBP decisions based on multiple criteria, including cost-effectiveness (with its shortcomings) – e.g. in Pakistan, Iran, Ethiopia. Especially in situations where MCDA is used for HBP design/revision, explicit criteria other than CE typically include feasibility (of implementation), cultural acceptability, equity concerns – which are difficult to quantify and thereby difficult to trade-off with quantitative data – but also quantifiable criteria such as budget impact. I am missing an acknowledgement that the inclusion of such criteria – along with CE – takes care, at least in part, of some of the forecasts listed in lines 13-23 as ‘ideally required’.
3. Lines 11-12, “… the appropriate design for HBPs is far more challenging [3,4]. This ideally requires, for each intervention considered for inclusion, an accurate forecast of: ….”: the five requirements listed here (i to v) are incomplete, they do not do justice to the systematic HBP design/revision approaches applied in other countries which involve the collection and appraisal of the available evidence on a predefined set of criteria.
4. Lines 29-31, “These estimates of CE are typically drawn from diverse geographical and temporal contexts, while a complete lack of available CE evidence on a significant number of interventions means that these must be excluded a priori from the analysis [5].”: the first part of this sentence is correct, the latter part is incorrect. Moreover it ignores the fact that many HBP design/revision trajectories include an appreciation of the strength of the available evidence (on CE and other criteria that are deemed important).
5. Lines 32-34, “The collated CE estimates are then considered for inclusion using a league table approach [1], constrained optimisation [8], or multi-criteria decision analysis (MCDA) [9]: Ref #9 is really an old one. There are much more recent sources. See for example: Baltussen et al. Multi-criteria decision analysis to support HTA agencies – benefits, limitations and the way forward. Value in Health 2019; 20(11):1283-8. Doi 10.1016/j.jval.2019.06.014 and Baltussen et al. The use of cost-effectiveness analysis for health benefit package design – should countries follow a sectoral, incremental or hybrid approach? Cost Eff Resour Alloc 2023;21(1):75. Doi 10.1186/s12962-023-00484-2.
6. Lines 57-63, on Thanzi La Onse (TLO), “… (it) is designed to comprehensively forecast, specifically in the context of Malawi, the health outcome achievable by any HBP while self-consistently modelling ii), making explicit — and hence easily modifiable — assumptions about iii) and iv), and enforcing healthcare-resource constraints. Crucially, the TLO simulation addresses the limitations of other HBP design methods outlined above by providing a country- specific predictive tool that sidesteps data extrapolation and intervention exclusion issues, while self-consistently accounting for feedback effects between interventions.” : what were the sources that informed these explicit assumptions in your study? Was it all desk work? I would be interested to know whether and, if so, how experienced Malawian hospital-based clinicians/service providers were involved in developing and testing the TLO simulation; I suggest this would merit a paragraph in the Methods section.
7. In that same paragraph, you claim that the TLO simulation “sidesteps … intervention exclusion issues” (also referred to in several other places in the paper). What exactly do you mean by this? Elsewhere in the paper an example is given of the complete exclusion of HIV treatment (lines 348-349; line 497), but I suspect that was a special case where other considerations may have come in (source of funding). Hence I’m not sure about the ‘intervention exclusion issue’, which you refer to several times in the Discussion section.
8. Methods, section 2.1 Overview of analytical approach, lines 76-77, “High-priority services or patients will be attended first, such that lower-priority ones will only be attended if healthcare workers still have time available to do so, …” : this assumes that available staff time is the single most important factor to consider for service providers (which service providers, more precisely?) to prioritise services. Isn’t that a huge assumption, which ignores the possibility of equipment breakdowns (e.g. radiology), stock-outs of medical products (partly dealt with in section 2.2), electricity cuts, dysfunctionality of patient transportation by ambulance – all not so rare phenomena that serious hinder health service provision in Malawi. Also, in HBO design/revision it’s not about service providers giving priority to one service over the other! I may miss the point that the authors try to make, but then would appreciate their response in a rebuttal.
9. Methods, section 2.2 Resource constraints, “Furthermore, the healthcare system is only allowed to dispense treatments until such capabilities are exhausted in a rigid mode of service delivery, characterised by the following assumptions: …”: while I appreciate that assumptions are inevitable, it appears that some of the assumptions might as well be considered limitations of the TLO simulation that may not necessarily weigh up against the disadvantages listed earlier of mainstream prioritisation approaches (incl MCDA). For example, (i) HSIs can only be delivered in the assumed expected duration given the treatment type and cannot be shortened by medical practitioners to accommodate a high demand for services on the day; (ii) No task shifting between medical cadres is allowed.

Minor comment:

10. Introduction, Lines 8-10, “HBPs seek to establish which services the public healthcare system should focus on delivering, often provided free-of-charge, given that IT is unable to take financial responsibility for the full range of possible services [2].”: grammatically ‘it’ refers to the public healthcare system. But not clear whether the latter part of the sentence (‘given that …’) refers to the need to focus on services, or to the free-of-charge aspect of provided services. Logically it is the focus, but then that requires a little rephrasing.
