# Supplementary material for: A new approach to Health Benefits Package design: an application of the Thanzi La Onse model in Malawi
Source: PLoS Comput Biol. 2024 Sep 30;20(9):e1012462. doi: 10.1371/journal.pcbi.1012462 (PMC11567512; doi:10.1371/journal.pcbi.1012462)
Supplement: Attachment — Submitted filename: PLOS review.docx [file pcbi.1012462.s007.docx]

Comments on the introductory sentence of abstract: “An optimized allocation of limited resources in low-income settings offers the opportunity to achieve important gains in health without requiring costly investments in health system capacity.” I do not agree that an optimized allocation of resources will necessarily (or even) result in gains in health without requiring costly investments in health system capacity. Neither do I agree that health system capacity investments are necessarily costly.

Line 5: I disagree with the use of the word “misallocation” in line 5. Perhaps nonideal allocation would be a better terminology: misallocation suggests fraudulent use, which is not investigated in this paper.

Lines 12-47: The process definition of health benefits package design, and the ensuing discussion, is problematic for 2 reasons: (a) it focuses almost entirely on curative services and does not take preventative services (which also reduce DALYs as it reduces disease burden) into account. (b) Cost effectiveness analyses are only 1 input into the process to determine a health benefits package: this article suggests that the outcome of a CE analysis process necessarily directly ‘translates’ into a HBP. This is a faulty assumption. Other program considerations also need to be taken into account, including the country’s health financing mechanisms, implementation modalities, and others. A country could, for example, have HIV as a main cause of disease burden but because the efforts are externally funded by the GF and PEPFAR, it is not included in a country’s HBP as a core set of services that the Government would provide. The cost effectiveness description provided in these paragraphs do not take the various modalities of CE into account: it also seems to be used interchangeably with HBP (cf line 41 ‘HBP strategy’). The 2 resources below are good places to look at both HBP design considerations and at CE options.

- <https://www.who.int/teams/health-systems-governance-and-financing/economic-analysis/health-technology-assessment-and-benefit-package-design/resource-guide-for-the-use-of-hta-and-hbp-design-processes/what-are-the-overall-principles-of-hbp-design>
- https://www.cgdev.org/publication/whats-in-whats-out-designing-benefits-universal-health-coverage

The write up reflects a certain simplicity / naivety about how HBP designs work. Because of these reasons, I would advise the authors to revise these paragraphs to focus on the different approaches for doing resource allocations in health (which is not only through CE analyses), and to write about how these approaches leads to the development of a HBP, as well as the other factors that influence a HBP design. The purpose of the model is to address optimal resource allocations in health and so this context will be most useful for readers of the article.

Lines 53-54: what is meant by ‘explicit HBP design’ (as opposed to implicit HBP design) and what is the reference for this?

Line 56: The acronym, EHP, needs to be defined.

Lines 67 – 82: for the reasons outlined here, it is problematic that the article’s context only refers to cost effectiveness analyses and not to the wider range of prioritization analyses methods that have been used to do prioritization in health (e.g. see here: <https://openknowledge.worldbank.org/entities/publication/5d90476c-d0ef-5a74-b770-25899018d8ed> ). The TLO model is a resource prioritization model – i.e. a model aimed at improving allocative efficiency. This is reiterated in other papers that talks of the importance of allocative efficiency dealing with what needs to be ‘more of and less of’ as opposed to ‘what is in and what is out.’ [cross referencing to the earlier point about lines 12-47, the authors again in this section assumes that a HBP is the sum total of what a health system finance, which is not the case: it is often that sub set of health interventions that will be covered by a national health insurance system]. It would make for a more powerful paper if the authors compared their TLO approach against all the other prioritization models in health financing. This will help others understand better what the TLO approach offers over and above other approaches. This handbook, compiled by the Joint Learning Network for Universal Health Coverage, clearly outlines the various approaches in health prioritization: <https://jointlearningnetwork.org/resources/health-priority-setting-a-practitioners-handbook/#:~:text=Regardless%20of%20their%20country's%20income,%2C%20the%20Philippines%2C%20and%20Vietnam>.

Line 83: Did they men ‘ practice’ as opposed to ‘practise’ ?

Lines 99 – 101: it would be important to defined the models of health seeking behavior that were included in the model and on which the assumptions are based.

Lines 105 – 108: What about below-facility costs, like the costs of community health workers? This was not referenced explicitly in this explanation, so not sure if this was included.

Lines 112 - 127: It was interesting to note that the deliberate decision was made to exclude task shifting. Since there are health resource prioritization efforts that have been attempted specifically through task shifting as a way to improve allocative efficiency, it would be good for the authors to explain why they decided to exclude task shifting. Also, why did the authors neither mention nor include strategic purchasing as ways to improve prioritization?

Lines 138 – 160: this figure and the ensuing explanation re-emphasizes what I believe is a problematic limitation and assumption of the model: it assumes that all healthcare gets provided at health facilities by healthcare providers and awards ‘service delivery slots’ based on the health system’s capacity to deliver such service slots, accordingly. (lines 196-199 also make this point, describing 82 treatments that patients can receive for their conditions (suggesting that this model focuses only on ‘sick-care’). It seems to exclude community health workers or self directed modes of service access for preventative purchases (like condom education for HIV prevention). This seems to create an overly medicalized model of curative service delivery and prioritization. The authors should indicate that they recognize this and explain why they made this decision in creating the model.

Lines 162 – 206: The focus on prioritisation of services and the people who need them, has been done in other allocative efficiency models too. It would be good for the authors to recognize other similar modelling approaches too.

Lines 217 – 219: reference these sources

Lines 236 – 267: In reviewing the results, what was not clear was the extent to which the reductions in DALY were significant in terms of the temporal trends in DALYs in Malawi. Using a reduction in DALYs as the main metric of the analysis outcomes s problematic as a single estimate, without the context of temporal DALY changes: In evaluating the 9% reduction in DALYs, it is important to understand the overall DALY trends in the years leading up to the period of reduction. According to the IHME DALY estimates, DALYs were already quite precipitously falling in Malawi between 1990 and 2019 -- <https://www.healthdata.org/research-analysis/health-by-location/profiles/malawi> -- so I am unsure of how to interpret the program and funding significance of the 9% reduction. Similarly, the strategy to focus on vulnerable patients “offers consistent health gains on a year-to-year basis” but it is not clear whether the health gains would have been consistent anyway, regardless of the approach to prioritization.

Lines 300 – 390: One of the results that the authors might want to add, is the remarkable similarity in temporal changes in overall disease burden, regardless of the prioritization approach chosen. In fact, this bores the question whether a conclusion should be that allocations based on cost effectiveness are not the only way in which to do prioritization. In fact, I think that it would be best to split this paper into 2 papers: one with the TLO results and a second that compares the TLO results to all the methods of prioritization, including to MCDA and other mathematical prioritization results. Else, the TLO results compared to a sub set of prioritization models, presents a skewed picture.

Lines 408-416: What I don’t understand about these results, is (a) the top ten causes of disase burden listed here is different than the top ten causes of disease burden as per the most authoritative estimates from the Institute of Health Metrics and Evaluation – see below …. available here https://www.healthdata.org/research-analysis/health-by-location/profiles/malawi

Whilst I did not look at the code or data used (it was not available for review), I am concerned that all the models presented in the paper shows a rapid increase in DALYs from 2023 to 2028 and then a flattening after that. I don’t understand the reasons for this as the overall disease burden in Malawi is decreasing, not increasing as dramatically as the model results all show.

**Other thoughts:**

It was surprising to me that the authors did not elaborate on the MCDA method of prioritization or use it as one of the comparisons, especially given that their method is a form of MCDA (with modelling added). A good reference is to start here: <https://www.ncbi.nlm.nih.gov/pmc/articles/PMC1560167/>
