# Supplementary material for: A new approach to Health Benefits Package design: an application of the Thanzi La Onse model in Malawi
Source: PLoS Comput Biol. 2024 Sep 30;20(9):e1012462. doi: 10.1371/journal.pcbi.1012462 (PMC11567512; doi:10.1371/journal.pcbi.1012462)
Supplement: Attachment — Submitted filename: Reply_to_referees.docx [file pcbi.1012462.s008.docx]

*We would like to thank the referees for taking the time to provide us with thorough and extensive feedback on the draft, which has undoubtedly allowed us to improve its quality, and which has given us interesting and important insights on the issues considered by our work.*

*In particular, in light of some of the referees’ comments, we have come to appreciate the limitations in the manuscript's original Introduction in making important distinctions between single-criterion quantitative analyses (e.g. CE-evidence based ones like LCOA) and the broader deliberative process in HBPs design, including the use of MCDA. We have therefore significantly revised the manuscript's Introduction to make this distinction with more clarity and to more clearly illustrate how our approach can contribute to the wider HBP generative process.*

*We further point out that upon checking all results prior resubmission we spotted a rounding error in the reported percentage improvement achieved from the LCOA policy, which is closer to ~8% than ~9% as previously reported. We have corrected this in the manuscript.*

*We provide detailed replies to each of the comments in more detail below.*

Referee A

The paper nicely lays out the shortcomings of cost-effectiveness data, rightly calls for modesty in generalising such data across settings and timeframes, and presents an interesting novel approach to health service prioritisation.

The structure of the paper is a bit atypical: usually the study objectives are placed at the end of the introduction (before the methods section), but in this paper the study aim comes at the end of the methods section (lines 230-234). I personally have no objection, and leave it to the Editor(s) to decide whether this is acceptable. I would however recommend a specification of the dual study aim (2x “We seek to …”) to better align them with the 4 subsections in Results.

*We thank the referee for suggesting improvements to the structure of the manuscript. Following the referee’s suggestion, we have:*

- *Included the study aims at the end of the Introduction;*
- *Restructured the Results section to better align with these aims;*

Also in relation to structure, section 3.3 (What makes a prioritisation strategy successful? – with 4 success factors) appears to be more something like a discussion. Please consider moving this from the Results section to Discussion.

*We believe this section is more appropriately included in the Results section, as it outlines the findings of one of the stated aims of the analysis (i.e. using these simulations to understand what aspects of a given policy more or less successful). We are however happy to follow the editor’s recommendation on this point.*

I suggest to shorten the Conclusions section. Repetition of things that have been stated earlier in the paper (which takes up the first half of this section) needs to be avoided in any case.

*We have dropped the first paragraph in Conclusions entirely, since this - as the referee pointed out -  mostly repeated what had already been stated elsewhere in the paper.*

My comments are as follows.

A1. Some strong statements are made in the Abstract, without sufficient supporting references in the paper. For instance,

(a) “Currently available methods to guide policymaking are limited and rely upon strong assumptions.” – it does not become clear (from the Introduction or Discussion) which methods are being referred to and which sources in particular justify this claim?

*The Introduction has been significantly revised to clarify which methods are referenced in the abstract and what their limitations are.*

 (b) “This strategy instead offers consistent health gains on a year-to-year basis and a 5% reduction in DALYs overall, which suggests a more patient-centred approach in future might prove beneficial.” – ‘instead’ of what? ‘More patient-centred’ than which other approach? The term ‘patient-centred’ may mislead the reader. I believe it’s important to add approach ‘to prioritisation’; or say ‘service prioritisation based on patient characteristics, rather than service types’.

*Following the referee’s comment, we have reformulated this sentence in the abstract as follows:*

*“We found that the HBP emerging from a linear constrained optimisation analysis (LCOA) achieved the largest health gain - a ~8\% reduction in disability adjusted life years (DALYs) between 2023 and 2042 compared to the benchmark scenario - by concentrating resources on high-impact treatments. This HBP however incurred a relative excess in DALYs in the first few years of its implementation. Other feasible approaches to prioritisation were assessed, including service prioritisation based on patient characteristics, rather than service type. Unlike the LCOA-based HBP, this approach achieved consistent health gains relative to the benchmark scenario on a year-to-year basis and a 5% reduction in DALYs over the whole period, which suggests an approach based upon patient characteristics might prove beneficial in the future.”*

A2. The paper does refer to commonly used methods and databases on the cost-effectiveness of health services/ interventions (such as DCP, TUFTS CE registry, WHO-CHOICE; the UHC Compendium would also deserve mentioning) but it doesn’t sufficiently acknowledge recent guidance documents and literature on country experiences with extensive stakeholder deliberation to inform HBP decisions based on multiple criteria, including cost-effectiveness (with its shortcomings) – e.g. in Pakistan, Iran, Ethiopia. Especially in situations where MCDA is used for HBP design/revision, explicit criteria other than CE typically include feasibility (of implementation), cultural acceptability, equity concerns – which are difficult to quantify and thereby difficult to trade-off with quantitative data – but also quantifiable criteria such as budget impact. I am missing an acknowledgement that the inclusion of such criteria – along with CE – takes care, at least in part, of some of the forecasts listed in lines 13-23 as ‘ideally required’.

*We have removed lines 13-23 entirely, and replaced them, as suggested by the referees, with a description of the wider deliberative process which can include CE analyses as one of its inputs.*

A3. Lines 11-12, “… the appropriate design for HBPs is far more challenging [3,4]. This ideally requires, for each intervention considered for inclusion, an accurate forecast of: ….”: the five requirements listed here (i to v) are incomplete, they do not do justice to the systematic HBP design/revision approaches applied in other countries which involve the collection and appraisal of the available evidence on a predefined set of criteria.

*We have removed the bullet points and replaced them with a description of the wider deliberative process undertaken by stakeholders to generate HBPs, as suggested by the referees.*

A4. Lines 29-31, “These estimates of CE are typically drawn from diverse geographical and temporal contexts, while a complete lack of available CE evidence on a significant number of interventions means that these must be excluded a priori from the analysis [5].”: the first part of this sentence is correct, the latter part is incorrect. Moreover it ignores the fact that many HBP design/revision trajectories include an appreciation of the strength of the available evidence (on CE and other criteria that are deemed important).

*We appreciate the point made here by the referee. What we meant in this sentence by exclusion from the “analysis” is not the ultimate exclusion for the final HBP, but rather from the quantitative, single-criterion metric analysis (e.g. LCOA) itself (see* [*https://gh.bmj.com/content/3/2/e000607*](https://gh.bmj.com/content/3/2/e000607) *and* [*https://onlinelibrary.wiley.com/doi/10.1002/hec.4664*](https://onlinelibrary.wiley.com/doi/10.1002/hec.4664)*). We have now stated so explicitly in the revised Introduction and highlighted that such issues can be overcome through participatory and deliberative processes (including use of MCDA). References on inclusion of uncertainty have also been included.*

A5. Lines 32-34, “The collated CE estimates are then considered for inclusion using a league table approach [1], constrained optimisation [8], or multi-criteria decision analysis (MCDA) [9]: Ref #9 is really an old one. There are much more recent sources. See for example: Baltussen et al. Multi-criteria decision analysis to support HTA agencies – benefits, limitations and the way forward. Value in Health 2019; 20(11):1283-8. Doi 10.1016/j.jval.2019.06.014 and Baltussen et al. The use of cost-effectiveness analysis for health benefit package design – should countries follow a sectoral, incremental or hybrid approach? Cost Eff Resour Alloc 2023;21(1):75. Doi 10.1186/s12962-023-00484-2.

*We thank the referee for suggesting more recent references, which we have added to the manuscript.*

A6. Lines 57-63, on Thanzi La Onse (TLO), “… (it) is designed to comprehensively forecast, specifically in the context of Malawi, the health outcome achievable by any HBP while self-consistently modelling ii), making explicit — and hence easily modifiable — assumptions about iii) and iv), and enforcing healthcare-resource constraints. Crucially, the TLO simulation addresses the limitations of other HBP design methods outlined above by providing a country- specific predictive tool that sidesteps data extrapolation and intervention exclusion issues, while self-consistently accounting for feedback effects between interventions.” : what were the sources that informed these explicit assumptions in your study? Was it all desk work? I would be interested to know whether and, if so, how experienced Malawian hospital-based clinicians/service providers were involved in developing and testing the TLO simulation; I suggest this would merit a paragraph in the Methods section.

*To avoid replicating information provided elsewhere, we opted not to include details of the model that are already presented in the model’s overview paper, whose open-source link was provided as a reference (https://www.medrxiv.org/content/10.1101/2024.01.04.24300834v1). We have now included the following sentence in the Methods section:*

*“Input from the Malawian Ministry of Health, as well as from several clinicians working in the country, was sought and accounted for at all development stages of the model, alongside a large number of data sources (see https://www.tlomodel.org/data_sources.html).”*

A7. In that same paragraph, you claim that the TLO simulation “sidesteps … intervention exclusion issues” (also referred to in several other places in the paper). What exactly do you mean by this? Elsewhere in the paper an example is given of the complete exclusion of HIV treatment (lines 348-349; line 497), but I suspect that was a special case where other considerations may have come in (source of funding). Hence I’m not sure about the ‘intervention exclusion issue’, which you refer to several times in the Discussion section.

*The “intervention exclusion issue” mentioned in line 62 refers to the exclusion a priori from quantitative analyses of interventions for which evidence is not available (which was discussed in the referee’s comment A4). This has now been revised and clarified in the Introduction. Elsewhere “intervention exclusion” refers to the exclusion of an intervention from the public healthcare provision, either in the hypothetical HBP/prioritisation policy under consideration (e.g. the exclusion of HIV treatment from the LCOA prioritisation policy), or in the actual country’s provision (line 595).*

A8.

(a) Methods, section 2.1 Overview of analytical approach, lines 76-77, “High-priority services or patients will be attended first, such that lower-priority ones will only be attended if healthcare workers still have time available to do so, …” : this assumes that available staff time is the single most important factor to consider for service providers (which service providers, more precisely?) to prioritise services. Isn’t that a huge assumption, which ignores the possibility of equipment breakdowns (e.g. radiology), stock-outs of medical products (partly dealt with in section 2.2), electricity cuts, dysfunctionality of patient transportation by ambulance – all not so rare phenomena that serious hinder health service provision in Malawi.

*The referee is right in pointing out that consumable availability, effects of equipment availability/breakdown/electricity cuts, and ambulance availability will play an additional part in hindering service delivery alongside lack of available medical cadres.*

*The effect of consumable availability is already extensively included in the model and calibrated to present-day, Malawi-specific data based on the work of Mohan et al. (*[*https://papers.ssrn.com/sol3/papers.cfm?abstract_id=4424341*](https://papers.ssrn.com/sol3/papers.cfm?abstract_id=4424341)*). Furthermore, its effect is self-consistently linked to the demand on HCWs’ time on which we place our overall constraints (e.g. unavailability of consumables may lead to patients’ conditions deteriorating and requiring further care, causing additional demand on HCWs).*

*We also note that, in cases where this data is available, disease modules may already include the observed effect of equipment unavailability (whether due to complete lack thereof, breakdown, or related power outages) in the probability of outcome for specific appointments: for example, while the availability of blood pressure equipment is not captured, the probability that a woman may receive blood pressure measurement during an antenatal care appointment is calibrated to reported rates of such checks in Malawi, allowing for the model to account for the effect of the equipment availability.*

*However, while the impact of these factors is accounted for in estimating the health burden, it is of course true that currently the optimisation analysis conducted only focuses on how to optimise the use of HCW resources, rather than all constraints regulated by the MoH budget. This point is addressed in section 4.3 of the manuscript.*

*Finally, service providers included in the model are public and publicly-contracted* [*Christian Health Association of Malawi*](https://cham.org.mw/) *(CHAM) hospitals.*

(b) Also, in HBO design/revision it’s not about service providers giving priority to one service over the other! I may miss the point that the authors try to make, but then would appreciate their response in a rebuttal.

*We address the issue raised by the referee in section 4.4 of the Discussion. We report below a few of the considerations made in this section.*

*Whereas a standard HBP approach considers only the inclusion or exclusion of services, our analysis considered a more nuanced formulation of the HBP, whereby services are assigned a relative priority as well as possibly being excluded altogether (as in the HSSP-III and LCOA policies). Although formulated differently, this analysis still allowed us to evaluate the possible consequences that concentrating resources on specific services may have on the overall health outcome of the population, while exposing the areas of care which would most benefit from a reallocation of resources away from high-profile ones. This is consistent with the broader purpose of the HBP in defining which services the healthcare system should prioritise given limited resources, and further better reflects the reality of service provision following the adoption of an HBP in many instances. Finally, this approach - unlike a “what’s in/what’s out” approach - allowed us to consider prioritisation strategies centred around patient characteristics (e.g. prioritising mothers and children), which is in line with the evolving approach of the Government of Malawi to prioritise resource allocation based on need and vulnerability as well as the nature of the service, making this a more relevant and tailored analysis for the particular country under consideration.*

*In addition, we highlight that the TLO model would be equally able to evaluate a “classic” HBP design based on a “what’s in/what’s out” approach without any prioritisation at all (as was in fact the case for the HSSP-III policy considered). All the benefits of a model-based approach showcased in this analysis (which are discussed in detail in the Introduction) could therefore be equally applied to a more “classic” HBP evaluation in the future.*

A9. Methods, section 2.2 Resource constraints, “Furthermore, the healthcare system is only allowed to dispense treatments until such capabilities are exhausted in a rigid mode of service delivery, characterised by the following assumptions: …”: while I appreciate that assumptions are inevitable, it appears that some of the assumptions might as well be considered limitations of the TLO simulation that may not necessarily weigh up against the disadvantages listed earlier of mainstream prioritisation approaches (incl MCDA). For example, (i) HSIs can only be delivered in the assumed expected duration given the treatment type and cannot be shortened by medical practitioners to accommodate a high demand for services on the day; (ii) No task shifting between medical cadres is allowed.

*We provide a reply to this comment in three parts: in the first, we provide motivation for why such assumptions, rather than being “inevitable”, are made carefully and deliberately and should be adopted to be consistent with our stated aim of optimising HCW time through prioritisation; in the second, we provide considerations on the sensitivity of the results of our analysis to these assumptions; in the third, we respond to whether such assumptions mean that the TLO-based approach may “not necessarily weigh up against the disadvantages listed earlier of mainstream prioritisation approaches”.*

*a) Practices of overtime and reducing the time taken in service delivery relative to expected clinical standards are at best non-ideal, and at worst overburden medical workers and put patients at risk. As discussed in section 4.1 “an optimal healthcare system should aim to maximise the care delivered without relying on the over-working of its medical workforce, or the reduction of time taken to deliver HSIs, to do so.” We therefore believe that the adoption of a rigid healthcare system assumption in this analysis is by definition required by the objective of an optimisation analysis in HCW time allocation, and therefore adopt it deliberately. Further considerations on this point are included in section 4.1 on the draft.*

*In addition, by preventing any task-shifting, we ensure that appointments are completed following standard task allocation as identified by Malawi's Human Resources for Health Strategic Plan (HRH SP) 2018–2022, which at present is the most reliable information for how tasks are allocated in the delivery of different types of appointments. It would be a different (though also valuable) analysis to examine the extent to which hypothetical task shifting practices could increase the net capabilities of the workforce to deliver services.*

*b) We acknowledge that a sudden transition from a system that allows overtime and the reduction of appointment duration to one that “protects” HCWs and patients alike from such practices is unlikely to occur in reality, and that this leads to an “artificial” increase in DALYs at the start of the period considered, mainly due to the increase in HIv/AIDS cases (see discussion in section 4.1). However, we believe we sufficiently contain this effect on the findings of our analysis by exclusively evaluating the performance of different prioritisation policies relative to a baseline scenario subject to the same constraints (the “No prioritisation policy” approach), and not in absolute terms. To a first approximation, it is in fact reasonable to assume that if HCWs generally try to do their best to accommodate as many non-emergency patients as possible on a given day by shortening appointment duration and potentially doing overtime, they will tend to do so for all patients and all services indiscriminately. (Recall that emergency appointments always have the highest possible priority across all policies, hence are not affected by such considerations). Because, under this assumption, all prioritisation policies would equally benefit from the practice of shortening appointment duration and overtime equally, excluding such practices does not affect their relative performance. Therefore, although we acknowledge that limiting these practices may inflate the overall health burden simulated in that period, we believe all policies should similarly “suffer” from the assumption of a rigid healthcare system, hence it is unlikely to significantly affect the relative performance of different prioritisation policies, which is the overriding focus of this analysis.*

*c) While it is true that our model contains assumptions around the practice of overtime/shortening of appointments/task-shifting, so do necessarily (albeit, implicitly) all other mainstream approaches which rely on evidence-based CE or intervention effectiveness estimates. This is because such estimates implicitly include the amount of overtime/shortening of appointments/task-shifting taking place in that setting (and indeed, consumable availability, equipment availability, and all other factors mentioned by the referee in comment #A8), which may or may not reflect those of the setting they are generalised to. The comparison of evidence for different interventions obtained from different settings may additionally introduce an inconsistency in these assumptions, and hence a bias in the comparison of the evidence in favour of different interventions.*

*More fundamentally, while these assumptions are implicit and hence fixed in observed CE estimates, a model-based approach gives us the flexibility to revise and adjust such assumptions at will while ensuring that their impact on the overall health outcome is captured self-consistently in any hypothetical scenario (e.g., in light of new task-shifting policies, restrictions on the amount of overtime doctors are allowed to perform, a significant expansion in the number of interventions considered for inclusion, or - referring back to factors highlighted in comment #8 - worsening of consumable/equipment/ambulance/electricity availability following e.g. catastrophic weather events, etc). As HBP design methods should assist policymakers in forecasting the expected health return in a constantly evolving environment, we believe this makes model-based approaches an important complementary approach to other evidence-based ones.*

Minor comment:

A10. Introduction, Lines 8-10, “HBPs seek to establish which services the public healthcare system should focus on delivering, often provided free-of-charge, given that IT is unable to take financial responsibility for the full range of possible services [2].”: grammatically ‘it’ refers to the public healthcare system. But not clear whether the latter part of the sentence (‘given that …’) refers to the need to focus on services, or to the free-of-charge aspect of provided services. Logically it is the focus, but then that requires a little rephrasing.

*We simplified the sentence to “HBPs seek to establish which services the public healthcare system should focus on delivering free-of-charge, given that it is unable to take financial responsibility for the full range of possible services [2].”*

Referee B

B1. Comments on the introductory sentence of abstract: “An optimized allocation of limited resources in low-income settings offers the opportunity to achieve important gains in health without requiring costly investments in health system capacity.” I do not agree that an optimized allocation of resources will necessarily (or even) result in gains in health without requiring costly investments in health system capacity. Neither do I agree that health system capacity investments are necessarily costly.

*We have clarified this sentence in the abstract by changing it to :"An efficient allocation of limited resources in low-income settings offers the opportunity to improve population-health outcomes given the available health-system capacity." We have also corrected a similar sentence in the conclusions.*

B2. Line 5: I disagree with the use of the word “misallocation” in line 5. Perhaps nonideal allocation would be a better terminology: misallocation suggests fraudulent use, which is not investigated in this paper.

*We agree with the referee, and have changed “misallocation” to “nonideal” in the text.*

B3. Lines 12-47: The process definition of health benefits package design, and the ensuing discussion, is problematic for 2 reasons:

(a) it focuses almost entirely on curative services and does not take preventative services (which also reduce DALYs as it reduces disease burden) into account.

*The process definition of HBP we provide, and the subsequent discussion doesn’t distinguish between preventative and curative services, but refers to both kinds of interventions indiscriminately. Indeed, all considerations presented could be applicable to both. We also emphasise here and in reply to a later comment (B11) that preventative services such as ITNs distribution, use of PrEP, and outreach initiatives in condom use for contraception/HIV prevention are indeed included in the model. The full list of services considered is given in the overview paper and also at* [*https://www.tlomodel.org/hsi_events.html*](https://www.tlomodel.org/hsi_events.html)*.*

(b) Cost effectiveness analyses are only 1 input into the process to determine a health benefits package: this article suggests that the outcome of a CE analysis process necessarily directly ‘translates’ into a HBP. This is a faulty assumption. Other program considerations also need to be taken into account, including the country’s health financing mechanisms, implementation modalities, and others. A country could, for example, have HIV as a main cause of disease burden but because the efforts are externally funded by the GF and PEPFAR, it is not included in a country’s HBP as a core set of services that the Government would provide. The cost effectiveness description provided in these paragraphs do not take the various modalities of CE into account: it also seems to be used interchangeably with HBP (cf line 41 ‘HBP strategy’). The 2 resources below are good places to look at both HBP design considerations and at CE options. · <https://www.who.int/teams/health-systems-governance-and-financing/economic-analysis/health-technology-assessment-and-benefit-package-design/resource-guide-for-the-use-of-hta-and-hbp-design-processes/what-are-the-overall-principles-of-hbp-design>

·https://www.cgdev.org/publication/whats-in-whats-out-designing-benefits-universal-health-coverage

The write up reflects a certain simplicity / naivety about how HBP designs work. Because of these reasons, I would advise the authors to revise these paragraphs to focus on the different approaches for doing resource allocations in health (which is not only through CE analyses), and to write about how these approaches leads to the development of a HBP, as well as the other factors that influence a HBP design. The purpose of the model is to address optimal resource allocations in health and so this context will be most useful for readers of the article.

*As discussed at the beginning of our reply, we do indeed agree with the referee that, in the original formulation of the introduction, the lack of clarity in distinguishing between HBP design methods and CE/MCDA analyses may have incorrectly suggested that “the outcome of a CE analysis process necessarily directly ‘translates’ into a HBP”. As suggested by the referee(s), we have thoroughly revised the Introduction to more clearly discuss how CE analyses constitute only one of the many inputs considered in the wider, multi-criteria, deliberative process. We believe that the revised Introduction will satisfy the referee’s concerns on this point and include therein the reference suggested by the referee.*

B4. Lines 53-54: what is meant by ‘explicit HBP design’ (as opposed to implicit HBP design) and what is the reference for this?

*The referee correctly points out that ‘explicit’ here was confusing, hence we have removed it from the sentence. References on LCOA/MCDA methods which informed Malawi’s HSSP  can be found within the HSSP references themselves, as now stated in the manuscript.*

B5. Line 56: The acronym, EHP, needs to be defined.

*We have corrected this by replacing the acronym with “HBP”.*

B6. Lines 67 – 82: for the reasons outlined here, it is problematic that the article’s context only refers to cost effectiveness analyses and not to the wider range of prioritization analyses methods that have been used to do prioritization in health (e.g. see here: https://openknowledge.worldbank.org/entities/publication/5d90476c-d0ef-5a74-b770-25899018d8ed ). The TLO model is a resource prioritization model – i.e. a model aimed at improving allocative efficiency. This is reiterated in other papers that talks of the importance of allocative efficiency dealing with what needs to be ‘more of and less of’ as opposed to ‘what is in and what is out.’ [cross referencing to the earlier point about lines 12-47, the authors again in this section assumes that a HBP is the sum total of what a health system finance, which is not the case: it is often that sub set of health interventions that will be covered by a national health insurance system]. It would make for a more powerful paper if the authors compared their TLO approach against all the other prioritization models in health financing. This will help others understand better what the TLO approach offers over and above other approaches. This handbook, compiled by the Joint Learning Network for Universal Health Coverage, clearly outlines the various approaches in health prioritization: <https://jointlearningnetwork.org/resources/health-priority-setting-a-practitioners-handbook/#:~:text=Regardless%20of%20their%20country's%20income,%2C%20the%20Philippines%2C%20and%20Vietnam>.

*The TLO model is not a “resource prioritisation model”, as it could equally evaluate a purely “what’s in/what’s out” HBP and a prioritisation policy as an input. Indeed, two of the policies considered in the analysis, HSSP-III and LCOA, already include a “hard” exclusion of services as would be suggested by “classic” HBP design approaches. In this particular analysis we have chosen to additionally allow for a degree of prioritisation among interventions included; considerations on how these results can inform HBP design is included in section 4.4, and in the reply to comment A8(b).*

*What distinguishes the TLO model from other analytical approaches - regardless of whether these take a “what’s in/what’s out” or a purely prioritisation-based approach - is its ability to address important limitations that affect equally analyses of either type, as extensively discussed in the introduction. These include the fact that in such analyses the incidence of relevant diseases and medical conditions is assumed to be independent; that important comorbidity effects and interactions in the availability of interventions are not captured, as well as possible shifts in the epidemiological context over time; issues in estimating potential benefits due to double counting; and other limitations linked to the generalisation of evidence to different settings. Fundamentally, the TLO model is a fully mechanistic representation of how the need for healthcare arises and the way in which available resources for healthcare are marshalled to deliver care and resulting health benefits – and this is indeed a significant contribution.*

*As such limitations impact quantitative analyses both in the case of HBP and prioritisation approach studies, we believe there is no need for us to restrict our discussion to prioritisation studies alone, as the innovation offered by our approach is of benefit to both. We have however now acknowledged the important work on this front by discussing and referencing these studies in the introduction.*

B7. Line 83: Did they men ‘ practice’ as opposed to ‘practise’ ?

*We did, and have corrected accordingly.*

B8. Lines 99 – 101: it would be important to defined the models of health seeking behavior that were included in the model and on which the assumptions are based.

*We have added in that sentence a reference to the overview paper of the model, where such models are discussed. For the benefit of the referee, we provide below the relevant references:*

- [*https://journals.plos.org/plosone/article?id=10.1371/journal.pone.0263650*](https://journals.plos.org/plosone/article?id=10.1371/journal.pone.0263650)
- [*https://onlinelibrary.wiley.com/doi/abs/10.1111/tmi.13499*](https://onlinelibrary.wiley.com/doi/abs/10.1111/tmi.13499)
- [*https://www.ajol.info/index.php/mmj/article/view/202965*](https://www.ajol.info/index.php/mmj/article/view/202965)

B9. Lines 105 – 108: What about below-facility costs, like the costs of community health workers? This was not referenced explicitly in this explanation, so not sure if this was included.

*Community health workers are indeed included in our analysis (please refer to the model’s overview paper, accessible at* [*https://www.medrxiv.org/content/10.1101/2024.01.04.24300834v1*](https://www.medrxiv.org/content/10.1101/2024.01.04.24300834v1)*). We now explicitly say so in the main text of the manuscript.*

B10. Lines 112 - 127: It was interesting to note that the deliberate decision was made to exclude task shifting. Since there are health resource prioritization efforts that have been attempted specifically through task shifting as a way to improve allocative efficiency, it would be good for the authors to explain why they decided to exclude task shifting. Also, why did the authors neither mention nor include strategic purchasing as ways to improve prioritization?

*The primary reason for deliberately excluding potential task-shifting (and strategic purchasing) from considerations at this stage was to limit the parameter space of potential optimisation interventions, in order to isolate and more easily interpret the effect of service prioritisation alone based on current and country-specific task allocation guidelines (see reply to comment A9 - point (a), second paragraph - for a discussion on the task allocation guidelines assumed in the model).*

B11. Lines 138 – 160: this figure and the ensuing explanation re-emphasizes what I believe is a problematic limitation and assumption of the model: it assumes that all healthcare gets provided at health facilities by healthcare providers and awards ‘service delivery slots’ based on the health system’s capacity to deliver such service slots, accordingly. (lines 196-199 also make this point, describing 82 treatments that patients can receive for their conditions (suggesting that this model focuses only on ‘sick-care’). It seems to exclude community health workers or self directed modes of service access for preventative purchases (like condom education for HIV prevention). This seems to create an overly medicalized model of curative service delivery and prioritization. The authors should indicate that they recognize this and explain why they made this decision in creating the model.

*The model does include community health workers (as now stated explicitly in the draft), and further allows for a variety of non-medical initiatives (such as access to outreach programmes on condom use for HIV prevention/contraception mentioned by the referee, use of PrEP, distribution of ITNs to prevent malaria infections, and many others) to impact the probability of individuals contracting relevant diseases and their subsequent behaviour. While resource constraints are enforced at the healthcare system level, the role of non-medical initiatives is therefore thoroughly recognised and captured by the model. (The term ‘healthcare’ in this context does not only mean to include care provided to patients who are currently unwell, and we have clarified this in the revised text: “The incidence of disease and disability among individuals in the TLO simulation, combined with assumed models for health-seeking behaviour (see model's overview paper \cite{Hallett2024} and references therein), determine the number of people seeking specific healthcare system interactions (HSIs) at each of the healthcare facilities modelled in the simulation on any given day.* ***Such HSIs can be both preventative and curative in nature, while the impact of a variety of non-medical initiatives (such as outreach programmes on condom use for HIV prevention/contraception, use of insecticide-treated bed nets, etc) is captured through its effect on the probability of individuals contracting relevant diseases and subsequently seeking care.****)”*

*Because the analysis focused on how the delivery of medical services could be optimised, considerations around the potential scale up of such outreach initiatives was not included at this stage (although their impact at current levels was still captured). The model would be able to consider such analyses in the future.*

B12. Lines 162 – 206: The focus on prioritisation of services and the people who need them, has been done in other allocative efficiency models too. It would be good for the authors to recognize other similar modelling approaches too.

*The “Clinically Vulnerable” prioritisation strategy described in the referenced lines does not focus on the “prioritisation of services and the people who need them” as suggested by the referee; rather it means that the patient’s individual characteristics help determine whether they will receive high-priority access to any type of service. For example, under the particular formulation of the policy we considered, an HIV-positive individual will receive prioritised access to any service/treatment they may request, whether for cancer, diabetes, etc. We believe this is an altogether different approach from the one discussed by the referee.*

B13. Lines 217 – 219: reference these sources

*References have been added to the text.*

B14. Lines 236 – 267: In reviewing the results, what was not clear was the extent to which the reductions in DALY were significant in terms of the temporal trends in DALYs in Malawi. Using a reduction in DALYs as the main metric of the analysis outcomes s problematic as a single estimate, without the context of temporal DALY changes: In evaluating the 9% reduction in DALYs, it is important to understand the overall DALY trends in the years leading up to the period of reduction. According to the IHME DALY estimates, DALYs were already quite precipitously falling in Malawi between 1990 and 2019 -- https://www.healthdata.org/research-analysis/health-by-location/profiles/malawi -- so I am unsure of how to interpret the program and funding significance of the 9% reduction. Similarly, the strategy to focus on vulnerable patients “offers consistent health gains on a year-to-year basis” but it is not clear whether the health gains would have been consistent anyway, regardless of the approach to prioritization.

*We would like to emphasise that the percentage reductions in DALYs reported are not expressed relative to the DALYs incurred at the start of the period considered. Rather, the reductions reported are expressed as relative to a baseline scenario in the same 2023-2040 time period (i.e. the health-outcome that would have been achieved in the same period if no prioritisation strategy - the “No Policy” scenario - had been implemented). Therefore, even if “DALYs were already quite precipitously falling”, what our analysis shows is that this trend could have been improved on by an additional 8% if, in addition to all existing efforts, a different HBP strategy was put into place. This is why we don’t think information on the overall trend is very informative, and the result is best expressed as a percentage change of the baseline scenario over the same period. We do provide the percentage in all the results, which does serve, we believe, to give a sense of “proportion” to the effects being reported.*

*With regards to the consistency of the health-gains when focusing on vulnerable patients, this is again expressed as relative to the baseline scenario evaluated in the same period.*

B15. Lines 300 – 390: One of the results that the authors might want to add, is the remarkable similarity in temporal changes in overall disease burden, regardless of the prioritization approach chosen. In fact, this bores the question whether a conclusion should be that allocations based on cost effectiveness are not the only way in which to do prioritization. In fact, I think that it would be best to split this paper into 2 papers: one with the TLO results and a second that compares the TLO results to all the methods of prioritization, including to MCDA and other mathematical prioritization results. Else, the TLO results compared to a sub set of prioritization models, presents a skewed picture.

*We highlight that “MCDA and other mathematical prioritization results” are already included in this analysis, namely through the LCOA policy, which directly translated LCOA outputs into a prioritisation strategy, and the HSSP-III HBP policy, in which “interventions were prioritised based on initial input from the LCOA, augmented by MCDA incorporating the following factors - severity of illness, effectiveness, poverty reduction, vulnerable populations, and level of care.”*

*In the manuscript we acknowledge that our analysis focuses on “evaluating [a] limited set of possible prioritisation policies,” and therefore does not make claims that this set exhausts all possible strategies. As quoted in our conclusions, we aimed to show how the TLO model can provide “a unique tool with which to test HBPs designed specifically for Malawi, providing detailed evaluations on the health gains that can be achieved through the relative prioritisation of services given realistic constraints in available human resources for health”, therefore emphasising that, rather than making any claims that our analysis can establish an “optimal” HBP,  this kind of analysis can offer an additional input in the evaluation of policies obtained through other analytic strategies.*

B16. Lines 408-416: What I don’t understand about these results, is (a) the top ten causes of disase burden listed here is different than the top ten causes of disease burden as per the most authoritative estimates from the Institute of Health Metrics and Evaluation – see below …. available here <https://www.healthdata.org/research-analysis/health-by-location/profiles/malawi>

*The calibration of the TLO simulation to all available data (including, but not limited to that from IHME) is extensively discussed in the model’s overview paper (*[*https://www.medrxiv.org/content/10.1101/2024.01.04.24300834v1*](https://www.medrxiv.org/content/10.1101/2024.01.04.24300834v1)*). Please note that the top 10 causes of DALYs shown in this draft represent forecasts for the future when relying on a range of hypothetical HBP implementations and “rigid healthcare system” assumptions (see reply to next comment for further discussion on this point) and therefore will necessarily deviate from current trends.*

B17. Whilst I did not look at the code or data used (it was not available for review), I am concerned that all the models presented in the paper shows a rapid increase in DALYs from 2023 to 2028 and then a flattening after that. I don’t understand the reasons for this as the overall disease burden in Malawi is decreasing, not increasing as dramatically as the model results all show.

*We start by apologising if, due to poor communication on our part, the referee didn’t have access to the code or data used while reviewing the manuscript. All code is open source, as is the data used to calibrate the model, and both can be accessed via the model’s webpage at* [*www.tlomodel.org*](http://www.tlomodel.org)*.*

*As previously mentioned, the model is extensively calibrated to Malawi-specific data up to the present day for all medical conditions included (please refer to the model’s overview paper for details on this:* [*https://www.medrxiv.org/content/10.1101/2024.01.04.24300834v1*](https://www.medrxiv.org/content/10.1101/2024.01.04.24300834v1)*). The period considered in this particular analysis (2023-2040), however, spans a long-term future which necessarily includes epidemiological shifts from currently observed trends, and which furthermore is hypothetical, in which i) a number of possible HBPs - which do not necessarily reflect the HBP currently implemented in Malawi - are implemented, and ii) a “rigid health-system” is assumed. Both factors will play a part in shaping the health burden incurred under these hypothetical futures, and in determining why this may not be readily extrapolated from currently observed trends.*

*In regards to ii) we remind the referee that a rigid healthcare system assumption ensures that no overtime and no rushing of appointments can take place (as well as no task-shifting from current guidelines). The main reason for the observed initial increase in DALYs over the period analysed is that we ensure these do not take place over the period considered, while in reality both practices are likely to take place in Malawi. In the long-term, the assumed lack of expansion of available capabilities (discussed in section 4.4) and population growth further aggravate this increase, as would be expected.*

*A detailed discussion on why a rigid healthcare system assumption was deliberately adopted in this analysis, and how its effect on the relative performance of the policies - on which this analysis focuses - is contained by all policies being equally affected by the rigid healthcare system assumption, is included in the answer to comment A9 (in particular paragraphs a) and b)).*

Other thoughts:

B18. It was surprising to me that the authors did not elaborate on the MCDA method of prioritization or use it as one of the comparisons, especially given that their method is a form of MCDA (with modelling added). A good reference is to start here: <https://www.ncbi.nlm.nih.gov/pmc/articles/PMC1560167/>

*MCDA is now extensively discussed and referenced in the new version of the introduction. Please also notice that we did use a MCDA-based design in our comparisons, in the form of Malawi’s HSSP-III (scenario HSSP-III HBP) - see Table 1, description of “HBP from Health Sector Strategic Plan III”: “The interventions were prioritised based on initial input from the LCOA, augmented by MCDA incorporating the following factors - severity of illness, effectiveness, poverty reduction, vulnerable populations, and level of care.”*
